# MOTS-c attenuates lung ischemia-reperfusion injury via MYH9-Dependent nuclear translocation and transcriptional activation of antioxidant genes

**DOI:** 10.1016/j.redox.2025.103681

**Published:** 2025-05-15

**Authors:** Xiangyu Li, Faliang Zhan, Guangfeng Qiu, Peng Lu, Zihao Shen, Yuanpu Qi, Minchao Wu, Mingyu Chu, Jia Feng, Ziang Wen, Xin Yao, Ao Wang, Wanjun Jin, Xiao Zhang, Junjie Liao, Jialin Zhang, Meijuan Song, Wei Wang, Xiaowei Wang

**Affiliations:** a Department of Cardiovascular Surgery, The First Affiliated Hospital with Nanjing Medical University, Nanjing, China; b Department of Cardiothoracic Surgery, Yili Friendship Hospital, Yining, China; c Department of Respiratory and Critical Care Medicine, Yili Friendship Hospital, Yining, China; d Department of Geriatrics, The First Affiliated Hospital with Nanjing Medical University, Nanjing, China; e Mazankowski Alberta Heart Institute, University of Alberta, Canada; f Department of Cardiovascular Surgery, The Affiliated Taizhou People's Hospital of Nanjing Medical University, Taizhou School of Clinical Medicine, Nanjing Medical University, Taizhou, China

**Keywords:** Acute respiratory distress syndrome, Cardiopulmonary bypass, Ischemia reperfusion injury, Oxidative stress, MOTS-c, MYH9, CK2A

## Abstract

Acute respiratory distress syndrome (ARDS) following cardiopulmonary bypass (CPB) is driven by oxidative stress during lung ischemia-reperfusion injury (LIRI). Mitochondrial-derived peptide MOTS-c has emerged as a regulator of mitochondrial-nuclear communication, yet its role in CPB-induced ARDS remains unclear. Here, we identify MOTS-c as a critical mediator of endothelial protection against LIRI through MYH9-dependent nuclear translocation and transcriptional activation of antioxidant genes. In rat LIRI models, endothelial cells exhibited the most significant MOTS-c upregulation, correlating with barrier preservation and reduced oxidative stress. Mechanistically, hypoxia-reoxygenation (HR) triggered reactive oxygen species (ROS)-dependent phosphorylation of MYH9 at Ser1943 via casein kinase II subunit alpha (CK2A), enabling MOTS-c binding to MYH9-γ-Actin complexes for nuclear transport. RNA sequencing (RNA-seq) combined with chromatin immunoprecipitation sequencing (ChIP-seq) revealed direct MOTS-c interaction with promoters of antioxidant genes (e.g., HMOX1, NQO1), which harbor antioxidant response elements (AREs). Clinically, serum MOTS-c increments within 24 h post-CPB (ΔMOTS-c) outperformed traditional biomarkers in predicting ARDS incidence, with multivariate models incorporating ΔMOTS-c achieving superior discriminative power (AUC = 0.885). Exogenous MOTS-c administration in rats attenuated lung injury by reducing oxidative damage, inflammation, and mortality, recapitulating endogenous protective mechanisms. Our findings establish MOTS-c as a dual-function molecule—acting via ROS-CK2A-MYH9 signaling to activate nuclear antioxidant defenses and serving as a prognostic biomarker for CPB-related complications. This study bridges mitochondrial dynamics, nuclear transcriptional regulation, and clinical outcomes, offering novel preventive avenues for IRI-associated pathologies.

## Graphical abstract

Graphic abstract. MOTS-c attenuates lung ischemia-reperfusion injury via MYH9-dependent nuclear translocation and transcriptional activation of antioxidant gene.

**Figure:**
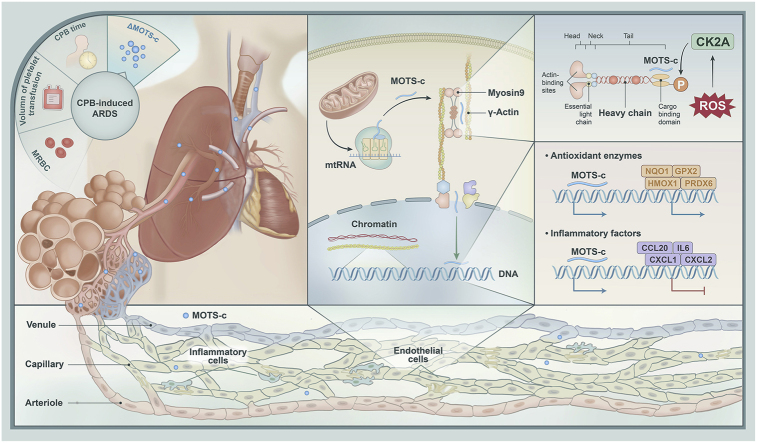

## 1 Introduction

Cardiopulmonary bypass (CPB) induces profound hemodynamic and physiologic perturbations, predisposing patients to various complications. Acute respiratory distress syndrome (ARDS), characterized by bilateral pulmonary infiltrates, severe hypoxemia, and non-cardiogenic edema, complicates 0.4–20 % of CPB procedures, with a mortality rate of more than 80 % [[1], [2], [3]]. The pathogenesis of CPB-induced ARDS is closely linked to lung ischemia-reperfusion injury (LIRI), which initiates oxidative stress, endothelial barrier dysfunction, and systemic inflammatory cascades [4]. The prevention of ARDS through optimized perioperative medical interventions is currently a common clinical strategy, encompassing both surgical techniques and patient preparation [5]. Surgical approaches include minimizing CPB duration, implementing heparin-coated circuits, and leukocyte filtration [[6], [7], [8], [9]]. Preoperative patient optimization involves strict smoking cessation, appropriate oxygen therapy, and respiratory training [[10], [11], [12], [13]]. Management of CPB-induced ARDS primarily focuses on respiratory support to optimize respiratory mechanics and facilitate recovery of injured lung tissue [14,15]. However, despite exhaustive clinical efforts, patients may still progress to severe ARDS. Notably, there remains a critical lack of methods to directly enhance pulmonary stress resistance, particularly in elderly high-risk populations. The development of such interventions holds significant clinical importance.

Mitochondria-derived peptides have emerged as critical regulators of cellular stress responses [16]. Among these, mitochondrial open reading frame of the 12S rRNA type-c (MOTS-c), is a 16-amino acid mitochondrial encoded peptide that was first discovered in 2015 by Cohen et al. [17]. Under physiological conditions, MOTS-c modulates homeostasis by coordinating mitochondrial-nuclear crosstalk, primarily through the AMP-activated protein kinase (AMPK) pathway, to avoid insulin resistance and metabolic dysregulation.

Emerging evidence highlights its broader roles in mitigating oxidative stress, inflammation, and mitochondrial dysfunction across diverse pathologies. Pyrroloquinoline quinone, a novel vitamin B, upregulated the mRNA and protein levels of MOTS-c in irradiated lung tissue and MLE-12 cells, effectively mitigating radiation-induced lung tissue damage, inflammation, oxidative stress, and epithelial cell apoptosis [18]. Long-term preventive and short-term therapeutic effects of MOTS-c treatments reverse nonalcoholic steatohepatitis (NASH)-induced mitochondrial metabolic deficiency and alleviate liver steatosis, cellular apoptosis, inflammation, and fibrosis [19]. In hepatitis B virus (HBV) infection, MOTS-c was found to promote mitochondrial biogenesis and enhance the MAVS signaling pathway [20]. Moreover, according to a study published in March 2025, remote ischemic preconditioning (RIPC) induces the expression of MOTS-c that mitigate LIRI by increasing Nrf2 protein levels [21].

The mitochondrial origin and clear biological role of MOTS-c make it a potential disease biomarker. Yaşar et al. observed that the MOTS-c levels was lower in the coronary artery disease (CAD) group and were a significant independent predictor of CAD in multiple regression analysis [22]. Plasma neuron-derived extracellular vesicles (NDEV) levels of MOTS-c are significantly lower in untreated patients with major depressive disorder (MDD) than in healthy controls and normalize in participants who responded to selective serotonin reuptake inhibitor (SSRI) therapy but remain persistently low in those who failed to respond [23]. A higher serum MOTS-c concentration is associated with greater muscle mass, force, and power generated during jumping in healthy individuals but not exercise capacity reflected by peak oxygen consumption [24]. However, its biological functions and clinical relevance in CPB-induced ARDS remain unexplored.

A mechanistic study published in *Cell Metabolism* in 2018 reveal that MOTS-c can undergo nuclear translocation in response to three different types of metabolic stress (glucose restriction, serum deprivation, and oxidative stress), facilitated by its hydrophobic core (_8_YIFY_11_), which may mediate protein interaction. Upon nuclear entry, MOTS-c activates antioxidant defense pathways via the _13_RKLR_16_ motif, directly upregulating genes containing antioxidant response elements (AREs), such as HMOX1 and NQO1 [25]. During the perioperative period of CPB cardiac surgery, the lung tissue undergoes ischemia-hypoxia and reperfusion oxidative stress, and whether MOTS-c can undergo nuclear translocation and play a nuclear biological role in this context aroused our curiosity. Moreover, the molecular partners governing MOTS-c nuclear translocation and its comprehensive transcriptional targets remain unidentified.

Non-muscle myosin II (NMII) is an actin-binding protein that comprises two heavy chains, two essential light chains and two regulatory light chains. In mammals, myosin heavy chain 9 (MYH9) is one of its component heavy chains and plays a critical role in cell adhesion and substance transport [26]. The N-terminal head portion of MYH9 is important for its ATPase function and actin binding, whereas the C-terminal rod domain and nonhelical tailpiece are critical for regulating filament formation and cargo binding [27,28]. Post-translational modification through phosphorylation, particularly within the tailpiece domain, serves as a critical regulatory mechanism for MYH9 functionality. Phosphorylation of the MYH9 at Ser1943 regulates single MYH9 molecule association with lytic granules to promote NK-cell cytotoxicity [29]. The onset of stimulated degranulation of RBL-2H3 mast cells coincides with the phosphorylation of MYH9 [30]. Current understanding of MYH9 phosphorylation mechanisms identifies casein kinase 2 (CK2) and protein kinase C (PKC) as principal regulatory kinases in vertebrates, with subsequent investigations predominantly concentrating on their functional characterization [27,31]. Wang et al. demonstrated that CK2 is the kinase responsible for the phosphorylation of MYH9 at Ser1943, allowing hMSC cytoskeleton to redistribute and play an important role in radiation-induced senescence [32]. CK2 but not PKC phosphorylated MYH9 from endothelial cells, thereby affecting angiotensin-converting enzyme (ACE) binding to MYH9 [33].

This study integrates molecular, translational, and clinical approaches to address these gaps. CPB-induced ARDS subjects pulmonary endothelial cells to metabolic perturbations, including hypoxia, serum deprivation, and glucose deficiency, which establish a permissive environment for MOTS-c nuclear translocation and genomic interaction. Building on this premise, we first establish MOTS-c's endothelial-specific upregulation and barrier-protective effects in LIRI models. Mechanistically, we identify MYH9 as a binding partner essential for ROS-CK2-dependent nuclear translocation of MOTS-c, enabling its transcriptional regulation of ARE-driven antioxidant and anti-inflammatory genes. Clinically, we demonstrate that dynamic MOTS-c increments within 24 h post-CPB correlate with reduced ARDS incidence and severity, outperforming traditional risk factors in predictive models. Finally, exogenous MOTS-c administration recapitulates endogenous protective effects in vivo, attenuating lung injury through conserved molecular pathways. Collectively, these findings position MOTS-c as both a prognostic biomarker and a preventive strategy for CPB-induced ARDS, bridging mitochondrial dynamics, nuclear transcriptional regulation, and clinical outcomes in critical care settings.

## 2 Results

### 2.1 MOTS-c is released into the bloodstream in patients after CPB and continues to rise over 24 h

**Fig. 1:**
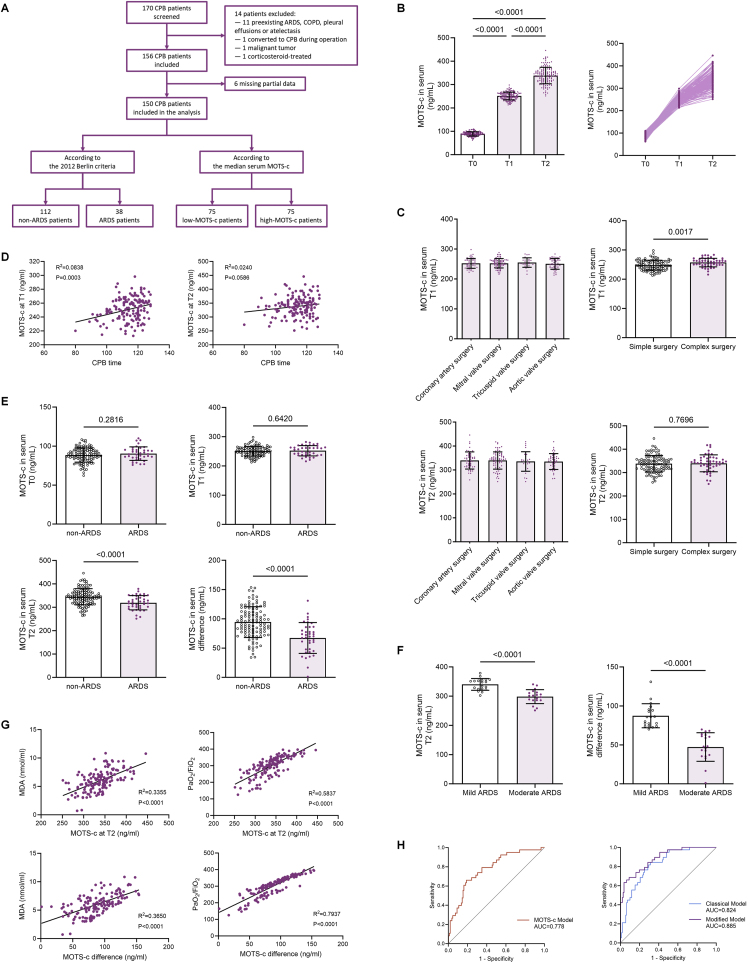
Sustained MOTS-c Release into Bloodstream Post-CPB Reflects Intraoperative and Postoperative Pathophysiological Processes **(A)** Schematic diagram of patient enrollment flow in the clinical study. **(B)** Serum MOTS-c concentrations at preoperative (T0), immediate postoperative (T1), and 24-h postoperative (T2) timepoints, along with dynamic changes across all enrolled patients (n = 150, one-way ANOVA post hoc Student-Newman-Keuls test). **(C)** Serum MOTS-c levels at T1 and T2 stratified by surgical type (coronary artery surgery, n = 52; mitral valve surgery, n = 76; tricuspid valve surgery, n = 28; aortic valve surgery, n = 46; simple surgery, n = 101; complex surgery, n = 49, one-way ANOVA post hoc Student-Newman-Keuls test). **(D)** Scatter plots with linear regression equations depicting the correlation between CPB duration and serum MOTS-c levels at T1 and T2 (n = 150, simple linear regression). **(E)** Serum MOTS-c concentrations at T0, T1, and T2, as well as the 24-h postoperative increment in MOTS-c levels (ΔMOTS-c = T2 – T1), stratified by the occurrence of ARDS within 72 h (non-ARDS, n = 112; ARDS, n = 38, two-tailed *t*-test). **(F)** Subgroup analysis of ARDS severity among ARDS patients, demonstrating T2 MOTS-c levels and ΔMOTS-c (mild ARDS, n = 19; moderate ARDS, n = 19, two-tailed *t*-test). **(G)** Scatter plots with linear regression equations illustrating the correlations of T2 MOTS-c levels and ΔMOTS-c with serum MDA concentrations and the lowest PaO_2_/FiO_2_ ratio within 72 h (n = 150, simple linear regression). **(H)** Receiver operating characteristic (ROC) curves for ARDS prediction using ΔMOTS-c alone, the classical model, and the modified model integrating ΔMOTS-c with intraoperative variables. Data are presented as the mean ± SEM. Abbreviations: CPB, cardiopulmonary bypass; ARDS, acute respiratory distress syndrome; COPD, chronic obstructive pulmonary disease.

The mitochondrial origin and diverse biological functions of MOTS-c make it a potential disease biomarker. We conducted a prospective clinical study in 150 patients undergoing CPB cardiac surgery to determine the relationship between MOTS-c levels and CPB-induced ARDS presence and severity. Arterial blood samples were collected preoperatively (T0), immediately post-CPB (T1), and 24 h postoperatively (T2) (Fig. 1A). Baseline characteristics are summarized in Table 1.

**Table 1:** Baseline characteristics of the study population (n = 150).

Characteristic	Value
Age, mean (SD), yr	61.7 (12.1)
Sex, Male, n (%)	85 (56.7)
BMI, mean (SD), kg/m^2^	24.6 (3.5)
EuroSCORE II, median (Q1–Q3)	2.9 (2.2–3.5)
NYHA, n (%)	
Ⅰ	14 (9.3)
Ⅱ	60 (40.0)
Ⅲ	62 (41.3)
Ⅳ	15 (10.0)
Smoking history, n (%)	71 (47.3)
Preoperative PaO_2_, mean (SD), mmHg	83.9 (13.0)
Preoperative PaCO_2_, mean (SD), mmHg	39.0 (6.7)
Comorbidities, n (%)	
Hypertension	98 (65.3)
Type 2 DM	56 (37.3)
Hyperlipidemia	8 (5.3)
Pior MI	14 (9.3)
Atrial fibrillation	30 (20.0)
Stroke	8 (5.3)
Peripheral vascular disease	8 (5.3)
Preoperative LVEF, mean (SD), %	53.8 (9.7)
Preoperative LVEDD, mean (SD), mm	55.9 (12.3)
Preoperative LAD, mean (SD), mm	38.0 (3.1)
Preoperative HR, mean (SD), bpm	69.6 (6.2)
Medications, n (%)	
Beta-blockers	99 (66.0)
RAASi	50 (33.3)
Statins	89 (59.3)
Diuretics	61 (40.7)
Calcium channel blockers	25 (16.7)
Aspirin	89 (59.3)
Preoperative laboratory parameters, median (Q1–Q3)	
Hs-cTnT, ng/L	15.2 (11.3–19.7)
NT-proBNP, ng/L	851.0 (226.1–2143.0)
Hemoglobin, g/L	130.0 (117.0–144.8)
WBC count, × 10^9^	5.8 (4.8–7.5)
Albumin, g/L	38.4 (35.8–41.3)
ALT, U/L	20.0 (13.0–32.5)
AST, U/L	24.0 (19.0–35.0)
Creatinine, μmol/L	76.0 (63.8–88.3)

Values are presented as mean ± standard deviation, median (Q1–Q3) or n (%).

Definition of abbreviations: ALT, alanine aminotransferase; AST, aspartate aminotransferase; BMI, body mass index; DM diabetes mellitus; EuroSCORE II, European System for Cardiac Operative Risk Evaluation II; HR, heart rate; Hs-cTnT, high-sensitivity cardiac troponin T; LAD, left atrial diameter; LVEDD, left ventricular end-diastolic dimension; LVEF, left ventricular ejection fraction; MI, myocardial infarction; NT-proBNP, N-Terminal Pro-Brain Natriuretic Peptide; NYHA, New York Heart Association grade for heart failure; Q1, quartile 1; Q3, quartile 3; RAASi, renin–angiotensin–aldosterone inhibitor; WBC, white blood cell.

Serum MOTS-c levels exhibited a significant postoperative surge, with median (IQR) concentrations rising from 89.3 (83.5–95.0) ng/mL at T0 to 250.7 (238.6–262.7) ng/mL at T1 (P < 0.001), peaking at T2 (339.4 [315.9–357.2] ng/mL) (Fig. 1B). Complex surgeries (multi-valve or combined valve/coronary procedures) showed higher T1 MOTS-c levels compared to simple procedures (257.4 [248.8–264.0] vs. 246.3 [235.6–261.1] ng/mL; P = 0.023), though this difference attenuated at T2 (Fig. 1C). Subgroup analyses by specific surgical types (coronary, mitral valve, tricuspid valve, aortic valve) revealed no intergroup differences at either timepoint (Fig. 1C).

CPB duration weakly correlated with T1 MOTS-c levels (R^2^ = 0.084, P = 0.0003), but not with T2 levels (R^2^ = 0.024, P = 0.059) (Fig. 1D). Preliminary results suggest that MOTS-c is released into the bloodstream in large quantities during CPB, and that the amount of release correlates with the duration of CPB but not with intraoperative cardiac manipulation. MOTS-c continued to be released despite 24 h of CPB shutdown and the patient's return to voluntary circulation, aligning with ongoing reperfusion injury observed in experimental models.

To elucidate time-dependent clinical relevance of serum MOTS-c levels, patients were stratified into high/low MOTS-c groups based on median T1 and T2 levels (Fig. 1A). Analysis of the preoperative baseline characteristics of the two groups at T1 or T2 did not yield much enlightening information. Patients with a preoperative history of statins or diuretics tended to have lower serum MOTS-c levels at T1, whereas patients with a preoperative history of rapid heart rate tended to have higher serum MOTS-c levels at T2 (Table 2). CPB-related variables and postoperative outcome indicators demonstrated significant patterns. Higher T1 MOTS-c levels correlated with prolonged CPB duration (P < 0.001), aortic clamp time (P = 0.002), and anesthesia time (P = 0.005), as well as increased intraoperative bleeding volume (P = 0.011), ultrafiltration (P = 0.008), and plasma transfusion (P = 0.003). When grouped according to T2 levels, the statistically significant differences in CPB- and transfusion-related variables were reduced to only ultrafiltration volume, cardioprotective fluid volume, and plasma transfusion volume. Low serum MOTS-c levels at T2 predicted adverse postoperative outcomes, including a 2.8-fold higher risk of ARDS within 72 h (P = 0.003), reduced oxygenation capacity (P < 0.001), prolonged mechanical ventilation (P = 0.008), and extended hospitalization (P = 0.012), whereas serum MOTS-c levels at T1 had no ability to predict these outcomes. These findings position T1 MOTS-c as a marker of intraoperative CPB stress and T2 MOTS-c as a prognostic indicator associated with pathophysiology and complications after CPB, particularly ARDS, highlighting its dual role in reflecting surgical burden and predicting clinical trajectories.

**Table 2:** Perioperative characteristics of low-MOTS-c and high-MOTS-c groups (n = 150).

Characteristic	T1-Low (n = 75)	T1-High (n = 75)	P	T2-Low (n = 75)	T2-High (n = 75)	P
Age, mean (SD), yr	62.1 (11.1)	61.2 (13.1)	0.668	64.4 (10.5)	58.9 (13.0)	**0.005**
Sex, Male, n (%)	43 (57.3)	42 (56.0)	0.869	45 (60.0)	40 (53.3)	0.410
BMI, mean (SD), kg/m^2^	24.3 (2.8)	24.9 (4.1)	0.257	24.5 (3.2)	24.9 (3.8)	0.487
EuroSCORE II, median (Q1–Q3)	3.1 (2.5–3.4)	2.8 (2.1–3.5)	0.303	2.9 (2.3–3.4)	3.1 (2.2–3.6)	0.993
NYHA, n (%)						
Ⅰ	10 (13.3)	4 (5.3)	0.092	8 (10.7)	6 (8.0)	0.575
Ⅱ	27 (36.0)	33 (44.0)	0.317	26 (34.7)	34 (45.3)	0.182
Ⅲ	33 (44.0)	29 (38.7)	0.507	36 (48.0)	26 (34.7)	0.097
Ⅳ	6 (8.0)	9 (12.0)	0.414	6 (8.0)	9 (12.0)	0.414
Smoking history, n (%)	36 (48.0)	35 (46.7)	0.870	38 (50.7)	33 (44.0)	0.414
Preoperative PaO_2_, mean (SD), mmHg	84.4 (14.1)	83.6 (11.9)	0.685	83.6 (13.2)	84.4 (13.0)	0.722
Preoperative PaCO_2_, mean (SD), mmHg	39.3 (6.6)	38.8 (6.9)	0.673	38.6 (6.8)	39.5 (6.7)	0.391
Comorbidities, n (%)						
Hypertension	48 (64.0)	50 (66.7)	0.731	50 (66.7)	48 (64.0)	0.731
Type 2 DM	22 (29.3)	34 (45.3)	0.043	25 (33.3)	31 (41.3)	0.311
Hyperlipidemia	5 (6.7)	3 (4.0)	0.467	5 (6.7)	3 (4.0)	0.467
Pior MI	5 (6.7)	9 (12.0)	0.262	8 (10.7)	6 (8.0)	0.575
Atrial fibrillation	16 (21.3)	14 (18.7)	0.683	13 (17.3)	17 (22.7)	0.414
Stroke	5 (6.7)	3 (4.0)	0.467	5 (6.7)	3 (4.0)	0.467
Peripheral vascular disease	4 (5.3)	4 (5.3)	1.000	5 (6.7)	3 (4.0)	0.467
Preoperative LVEF, mean (SD), %	52.9 (9.5)	54.8 (10.0)	0.223	53.3 (9.3)	54.3 (10.2)	0.538
Preoperative LVEDD, mean (SD), mm	57.2 (11.7)	54.7 (12.9)	0.225	56.8 (11.9)	55.1 (12.7)	0.401
Preoperative LAD, mean (SD), mm	38.0 (3.1)	38.0 (3.2)	0.914	38.1 (2.9)	37.8 (3.2)	0.516
Preoperative HR, mean (SD), bpm	69.6 (5.7)	69.6 (6.7)	0.991	68.2 (6.2)	71.0 (6.0)	**0.005**
Medications, n (%)						
Beta-blockers	50 (66.7)	46 (61.3)	0.496	47 (62.7)	49 (65.3)	0.734
RAASi	29 (38.7)	21 (28.0)	0.166	27 (36.0)	23 (30.7)	0.488
Statins	50 (66.7)	38 (50.7)	**0.047**	44 (58.7)	44 (58.7)	1.000
Diuretics	37 (49.3)	25 (33.3)	**0.047**	36 (48.0)	26 (34.7)	0.097
Calcium channel blockers	9 (12.0)	15 (20.0)	0.181	12 (16.0)	12 (16.0)	1.000
Aspirin	50 (66.7)	39 (52.0)	0.067	49 (65.3)	40 (53.3)	0.135
Preoperative laboratory parameters, median (Q1–Q3)						
Hs-cTnT, ng/L	14.8 (11.2–18.8)	15.3 (11.7–21.0)	0.581	14.7 (10.7–19.4)	16.2 (12.1–21.9)	0.423
NT-proBNP, ng/L	900.0 (196.2–2711.0)	750.9 (231.6–1876.0)	0.618	868.5 (216.8–1853.1)	726.6 (226.1–2464.0)	0.699
Hemoglobin, g/L	130.0 (116.0–147.0)	131.0 (117.0–142.0)	0.437	131.0 (116.0–147.0)	130.0 (117.0–142.0)	0.535
WBC count, × 10^9^	5.7 (4.6–8.0)	5.8 (4.9–7.0)	0.594	5.5 (4.5–7.2)	5.9 (5.0–7.5)	0.116
Albumin, g/L	38.7 (35.9–41.7)	38.2 (35.2–40.9)	0.136	38.0 (35.9–41.0)	38.6 (35.0–42.1)	0.863
ALT, U/L	19.0 (12.0–29.0)	22.0 (13.0–36.0)	0.148	20.7 (14.0–34.0)	20.0 (12.0–31.0)	0.589
AST, U/L	23.0 (19.0–29.1)	25.0 (19.0–40.0)	0.939	25.0 (20.0–35.0)	22.0 (17.0–35.0)	0.580
Creatinine, μmol/L	74.0 (60.0–85.8)	77.0 (68.5–91.5)	0.763	76.0 (69.0–90.0)	75.0 (62.0–87.0)	0.492
CPB-related variables						
Complex surgery, n (%)	22 (29.3)	27 (36.0)	0.384	23 (30.7)	26 (34.7)	0.601
Bleeding volume, median (Q1–Q3), ml	700.0 (500.0–900.0)	800.0 (700.0–900.0)	**0.042**	800.0 (600.0–900.0)	800.0 (600.0–900.0)	0.486
Input volume, median (Q1–Q3), ml	1730.0 (1580.0–1960.0)	1810.0 (1670.0–1970.0)	0.402	1780.0 (1580.0–1960.0)	1790.0 (1670.0–2000.0)	0.188
Anesthesia time, median (Q1–Q3), min	275.0 (251.0–316.0)	295.0 (271.0–321.0)	**0.042**	282.0 (255.0–319.0)	286.0 (259.0–319.0)	0.558
CPB time, median (Q1–Q3), min	113.0 (104.0–120.0)	117.0 (111.0–122.0)	**0.000**	113.0 (108.0–120.0)	116.0 (109.0–122.0)	0.064
Aortic cross-clamp time, median (Q1–Q3), min	82.0 (75.0–89.0)	88.0 (81.0–92.0)	**0.001**	84.0 (76.0–89.0)	86.0 (78.0–92.0)	0.106
Ultrafiltration volume, median (Q1–Q3), ml	2240.0 (2110.0–2320.0)	2600.0 (2510.0–2760.0)	**0.000**	2650.0 (2420.0–2910.0)	2370.0 (2270.0–2570.0)	**0.000**
Precharge fluid volume, median (Q1–Q3), ml	1080.0 (1070.0–1120.0)	1100.0 (1070.0–1120.0)	0.282	1090.0 (1070.0–1120.0)	1090.0 (1060.0–1120.0)	0.883
Additional fluid volume, median (Q1–Q3), ml	0.0 (0.0–134.0)	0.0 (0.0–115.5)	0.411	0.0 (0.0–130.6)	0.0 (0.0–122.2)	0.920
Cardioprotective fluid volume, median (Q1–Q3), ml	1800.0 (1800.0–1900.0)	1700.0 (1600.0–1800.0)	**0.000**	1800.0 (1800.0–1900.0)	1700.0 (1600.0–1700.0)	**0.000**
Urine volume, median (Q1–Q3), ml	1530.0 (1385.0–1657.0)	1461.0 (1369.0–1601.0)	0.237	1498.0 (1353.0–1646.0)	1487.0 (1385.0–1602.0)	0.894
Blood transfusion						
Transfusion reaction, n (%)	8 (10.7)	7 (9.3)	0.785	4 (5.3)	11 (14.7)	0.057
Crystalloid solution volume, median (Q1–Q3), ml	2500.0 (2420.0–2620.0)	2460.0 (2340.0–2640.0)	0.464	2490.0 (2370.0–2640.0)	2460.0 (2340.0–2600.0)	0.237
Volume of RBC transfusion, median (Q1–Q3), ml	4.3 (3.8–4.8)	4.0 (3.4–4.4)	**0.002**	4.2 (3.5–4.7)	4.1 (3.6–4.5)	0.654
Volume of plasma transfusion, median (Q1–Q3), ml	454.7 (426.8–479.8)	516.0 (495.0–550.1)	**0.000**	466.0 (437.9–487.6)	502.8 (477.2–542.9)	**0.000**
Volume of platelet transfusion, median (Q1–Q3), ml	0.0 (0.0–0.0)	0.0 (0.0–0.0)	0.793	0.0 (0.0–0.0)	0.0 (0.0–0.0)	0.189
MRBC, n (%)	31 (41.3)	31 (41.3)	1.000	35 (46.7)	27 (36.0)	0.185
Outcomes						
ARDS within 72 h, n (%)	18 (24.0)	20 (26.7)	0.707	27 (36.0)	11 (14.7)	**0.003**
Minimum PaO_2_/FiO_2_ ratio within 72 h, median (Q1–Q3)	303.9 (262.6–339.3)	309.5 (257.3–353.1)	0.818	275.9 (229.9–302.2)	340.4 (310.8–363.5)	**0.000**
Mechanical ventilation, median (Q1–Q3), hours	24.0 (23.0–25.0)	24.0 (23.0–25.0)	0.816	25.0 (24.0–26.0)	23.0 (22.0–24.0)	**0.000**
Incidence of AKI, n (%)	14 (18.7)	18 (24.0)	0.425	16 (21.3)	16 (21.3)	1.000
Incidence of Stroke, n (%)	1 (1.3)	0 (0.0)	0.316	1 (1.3)	0 (0.0)	0.316
New onset of Atrial fibrillation, n (%)	13 (17.3)	17 (22.7)	0.414	19 (25.3)	11 (14.7)	0.102
Length of ICU stay, median (Q1–Q3), hours	48.0 (43.0–52.0)	47.0 (43.0–51.0)	0.967	48.0 (44.0–52.0)	47.0 (42.0–51.0)	0.116
Length of hospital stay, median (Q1–Q3), days	18.0 (17.0–19.0)	18.0 (17.0–19.0)	0.435	19.0 (18.0–20.0)	17.0 (16.0–18.0)	**0.000**
In-hospital mortality, n (%)	3 (4.0)	0 (0.0)	0.080	1 (1.3)	2 (2.7)	0.560

Values are presented as mean ± standard deviation, median (Q1–Q3) or n (%).

Definition of abbreviations: AKI, acute kidney injury; ALT, alanine aminotransferase; ARDS, acute respiratory distress syndrome; AST, aspartate aminotransferase; BMI, body mass index; CPB, cardiopulmonary bypass; DM diabetes mellitus; EuroSCORE II, European System for Cardiac Operative Risk Evaluation II; HR, heart rate; Hs-cTnT, high-sensitivity cardiac troponin T; ICU, intensive care unit; LAD, left atrial diameter; LVEDD, left ventricular end-diastolic dimension; LVEF, left ventricular ejection fraction; MI, myocardial infarction; MRBC, massive transfusion of red blood cells; NT-proBNP, N-Terminal Pro-Brain Natriuretic Peptide; NYHA, New York Heart Association grade for heart failure; Q1, quartile 1; Q3, quartile 3; RAASi, renin–angiotensin–aldosterone inhibitor; RBC, red blood cell; WBC, white blood cell.

### 2.2 MOTS-c increment within 24 h after CPB is associated with ARDS

As Table 2 demonstrates, patients with lower serum MOTS-c levels at 24 h after CPB had a higher incidence of ARDS within 72 h, prompting us to further analyze the correlation between the two in depth. Patients were categorized into a postoperative ARDS group and a postoperative non-ARDS group based on their lowest oxygenation index within 72 h and imaging findings with reference to the 2012 ARDS Berlin criteria (Fig. 1A). Table 3 shows the perioperative characteristics of non-ARDS group (n = 112) and ARDS group (n = 38). ARDS patients had prolonged anesthesia duration (301.5 [273.0–327.3] vs. 281.0 [254.0–316.0] min; P = 0.011), extended CPB time (120.0 [115.8–123.0] vs. 113.0 [106.3–118.8] min; P < 0.001), prolonged aortic cross-clamp time (88.5 [84.0–92.3] vs. 83.0 [75.0–89.0] min; P = 0.001), more volume of platelet transfusion (0.0 [0.0–0.6] vs. 0.0 [0.0–0.0] mL; P < 0.001) and higher rates of massive red blood cell transfusion (63.2 % vs. 33.9 %; P = 0.002) compared to non-ARDS patients (Table 3). However, no intergroup differences were observed in average age, smoking history, type 2 diabetes mellitus, and preoperative left ventricular function (Table 3).

**Table 3:** Perioperative characteristics of non-ARDS and ARDS groups (n = 150).

Characteristic	non-ARDS (n = 112)	ARDS (n = 38)	P
Age, mean (SD), yr	60.8 (12.5)	64.3 (10.8)	0.123
Sex, Male, n (%)	66 (58.9)	19 (50.0)	0.337
BMI, mean (SD), kg/m^2^	24.7 (3.5)	24.5 (3.4)	0.679
EuroSCORE II, median (Q1–Q3)	3.0 (2.3–3.5)	2.8 (2.0–3.4)	0.202
NYHA, n (%)			
Ⅰ	12 (10.7)	2 (5.3)	0.318
Ⅱ	47 (42.0)	13 (34.2)	0.399
Ⅲ	43 (38.4)	19 (50.0)	0.209
Ⅳ	10 (8.9)	5 (13.2)	0.453
Smoking history, n (%)	52 (46.4)	19 (50.0)	0.703
Preoperative PaO_2_, mean (SD), mmHg	84.5 (13.0)	82.5 (13.0)	0.403
Preoperative PaCO_2_, mean (SD), mmHg	39.2 (6.7)	38.4 (6.8)	0.519
Comorbidities, n (%)			
Hypertension	71 (63.4)	27 (71.1)	0.391
Type 2 DM	40 (35.7)	16 (42.1)	0.482
Hyperlipidemia	6 (5.4)	2 (5.3)	0.982
Pior MI	10 (8.9)	4 (10.5)	0.770
Atrial fibrillation	21 (18.8)	9 (23.7)	0.511
Stroke	7 (6.3)	1 (2.6)	0.391
Peripheral vascular disease	6 (5.4)	2 (5.3)	0.982
Preoperative LVEF, mean (SD), %	54.2 (10.4)	52.7 (7.5)	0.400
Preoperative LVEDD, mean (SD), mm	55.8 (12.7)	56.3 (11.3)	0.856
Preoperative LAD, mean (SD), mm	38.0 (3.0)	38.0 (3.4)	0.994
Preoperative HR, mean (SD), bpm	69.9 (6.2)	68.7 (6.3)	0.282
Medications, n (%)			
Beta-blockers	71 (63.4)	25 (65.8)	0.790
RAASi	38 (33.9)	12 (31.6)	0.791
Statins	69 (61.6)	19 (50.0)	0.209
Diuretics	47 (42.0)	15 (39.5)	0.788
Calcium channel blockers	17 (15.2)	7 (18.4)	0.638
Aspirin	65 (58.0)	24 (63.2)	0.579
Preoperative laboratory parameters, median (Q1–Q3)			
Hs-cTnT, ng/L	15.9 (11.8–25.2)	13.4 (10.1–16.6)	0.174
NT-proBNP, ng/L	810.1 (225.4–2757.2)	875.5 (216.1–1797.5)	0.124
Hemoglobin, g/L	130.0 (116.0–142.0)	134.0 (120.0–146.0)	0.373
WBC count, × 10^9^	5.7 (4.9–7.5)	6.1 (4.2–8.3)	0.815
Albumin, g/L	38.6 (35.4–41.5)	38.0 (35.9–41.3)	0.780
ALT, U/L	20.0 (13.0–32.2)	21.4 (12.0–33.0)	0.716
AST, U/L	24.0 (19.0–35.0)	24.0 (18.5–32.8)	0.979
Creatinine, μmol/L	75.0 (63.0–90.9)	78.0 (63.0–87.3)	0.460
CPB-related variables			
Complex surgery, n (%)	35 (31.3)	14 (36.8)	0.525
Bleeding volume, median (Q1–Q3), ml	600.0 (600.0–700.0)	600.0 (500.0–700.0)	0.277
Input volume, median (Q1–Q3), ml	1775.0 (1645.0–1950.0)	1890.0 (1580.0–2047.5)	0.369
Anesthesia time, median (Q1–Q3), min	281.0 (254.0–316.0)	301.5 (273.0–327.3)	**0.011**
CPB time, median (Q1–Q3), min	113.0 (106.3–118.8)	120.0 (115.8–123.0)	**0.000**
Aortic cross-clamp time, median (Q1–Q3), min	83.0 (75.0–89.0)	88.5 (84.0–92.3)	**0.001**
Ultrafiltration volume, median (Q1–Q3), ml	2440.0 (2225.0–2567.5)	2440.0 (2247.5–2702.5)	0.278
Precharge fluid volume, median (Q1–Q3), ml	1090.0 (1070.0–1120.0)	1080.0 (1067.5–1120.0)	0.869
Additional fluid volume, median (Q1–Q3), ml	0.0 (0.0–123.5)	0.0 (0.0–130.4)	0.806
Cardioprotective fluid volume, median (Q1–Q3), ml	1800.0 (1700.0–1800.0)	1750.0 (1700.0–1800.0)	0.245
Urine volume, median (Q1–Q3), ml	1500.0 (1378.5–1643.5)	1486.0 (1373.0–1644.3)	0.861
Blood transfusion			
Transfusion reaction, n (%)	11 (9.8)	4 (10.5)	0.900
Crystalloid solution volume, median (Q1–Q3), ml	2515.0 (2375.0–2640.0)	2440.0 (2340.0–2545.0)	0.070
Volume of RBC transfusion, median (Q1–Q3), ml	4.1 (3.6–4.5)	4.4 (3.6–4.7)	0.272
Volume of plasma transfusion, median (Q1–Q3), ml	481.9 (452.5–514.4)	494.2 (465.4–530.4)	0.058
Volume of platelet transfusion, median (Q1–Q3), ml	0.0 (0.0–0.0)	0.0 (0.0–0.6)	**0.000**
MRBC, n (%)	38 (33.9)	24 (63.2)	**0.002**
Serum MOTS-c, median (Q1–Q3), ng/ml			
T0	89.2 (83.3–94.7)	89.7 (84.8–95.2)	0.282
T1	250.5 (239.2–262.2)	256.2 (237.7–264.7)	0.642
T2	344.8 (321.9–365.4)	319.9 (300.2–348.2)	**0.000**
D (T2 - T1)	94.2 (77.0–112.5)	69.9 (47.1–85.9)	**0.000**

Values are presented as mean ± standard deviation, median (Q1–Q3) or n (%).

Definition of abbreviations: AKI, acute kidney injury; ALT, alanine aminotransferase; ARDS, acute respiratory distress syndrome; AST, aspartate aminotransferase; BMI, body mass index; CPB, cardiopulmonary bypass; DM diabetes mellitus; EuroSCORE II, European System for Cardiac Operative Risk Evaluation II; HR, heart rate; Hs-cTnT, high-sensitivity cardiac troponin T; LAD, left atrial diameter; LVEDD, left ventricular end-diastolic dimension; LVEF, left ventricular ejection fraction; MI, myocardial infarction; MRBC, massive transfusion of red blood cells; NT-proBNP, N-Terminal Pro-Brain Natriuretic Peptide; NYHA, New York Heart Association grade for heart failure; Q1, quartile 1; Q3, quartile 3; RAASi, renin–angiotensin–aldosterone inhibitor; RBC, red blood cell; WBC, white blood cell.

Preoperative MOTS-c levels and T1 levels did not differ between groups, whereas postoperative ARDS patients demonstrated significantly lower T2 MOTS-c levels (319.9 [300.2–348.2] vs. 344.8 [321.9–365.4] ng/mL; P < 0.001) (Fig. 1E). To isolate CPB-specific effects, we calculated and introduced the difference in MOTS-c levels at T2 and T1 (ΔMOTS-c = T2 – T1), a variable that reflects the 24-h MOTS-c increment after CPB and correlates with the T1 and T2 levels at the same time (Table 3). ARDS patients showed markedly reduced ΔMOTS-c (69.9 [47.1–85.9] vs. 94.2 [77.0–112.5] ng/mL; P < 0.001) (Fig. 1E). Further validation of subgroup analysis for ARDS patients revealed that both the absolute T2 level and ΔMOTS-c decreased with increasing severity of ARDS patients after CPB, suggesting that MOTS-c correlates with ARDS severity (Fig. 1F).

Linear regression analysis revealed robust and clinically meaningful associations between MOTS-c and key pathophysiological markers. Specifically, T2 levels exhibited an inverse correlation with serum MDA levels (R^2^ = 0.336, P < 0.001) and a positive correlation with the 72-h lowest PaO_2_/FiO_2_ (R^2^ = 0.584, P < 0.001) (Fig. 1G). Notably, ΔMOTS-c surpassed static T2 measurements in predictive capacity, stronger correlations with both oxidative stress (R^2^ = 0.365 vs. 0.336) and oxygenation capacity (R^2^ = 0.794 vs. 0.584). These results underscores the unique value of ΔMOTS-c as a dynamic biomarker capturing dynamic changes in MOTS-c release during the critical 24-h postoperative window, offering enhanced precision in evaluating oxidative stress burden and lung injury severity. Consistent with experimental models of oxidative stress-induced MOTS-c release, these findings position ΔMOTS-c as a clinically actionable tool for ARDS risk stratification and post-CPB recovery monitoring.

### 2.3 MOTS-c increment within 24 h has superior predictive efficacy for CPB-induced ARDS

Current evidence regarding risk factors for postoperative ARDS remains conflicting, with no established consensus. Low ability to release MOTS-c in CPB-induced ARDS patients suggests that early postoperative MOTS-c absolute levels and dynamic increments may serve as promising biomarkers for predicting postoperative ARDS. As summarized in Table 3, univariate logistic regression analysis identified anesthesia time, CPB time, aortic cross-clamp time, volume of platelet transfusion, and MRBC as significant factors associated with ARDS (P < 0.05). To identify independent risk factors, these variables were subsequently incorporated into multivariate logistic regression analyses, both with and without the inclusion of T2 MOTS-c levels and ΔMOTS-c (Table 4).

**Table 4:** Multivariable logistic regression model using clinical characteristic for prediction of ARDS without (Classical model) and with (Modified model) serum MOTS-c.

Characteristic	Classical model		Modified model		
	Odds Ratio	P	Odds Ratio	P	
Anesthesia time^a^	0.998 (0.985–1.011)	0.721	0.996 (0.981–1.012)	0.659	
CPB time	1.219 (1.073–1.384)	**0.002**	1.261 (1.087–1.462)	**0.002**	
Aortic cross-clamp time^a^	0.934 (0.850–1.027)	0.158	0.933 (0.838–1.039)	0.206	
Volume of platelet transfusion	7.156 (1.930–26.529)	**0.003**	5.313 (1.294–21.819)	**0.020**	
MRBC	3.048 (1.244–7.472)	**0.015**	4.011 (1.358–11.845)	**0.012**	
Serum MOTS-c at T2^a^	Not included	NA	1.004 (0.973–1.037)	0.783	
Serum MOTS-c difference (T2 - T1)	Not included	NA	0.945 (0.906–0.985)	**0.008**	
Comparison of models					
AUC	0.824 (0.754–0.895)	<0.001	0.885 (0.825–0.945)	<0.001	
Difference of AUC	0.057 (0.004–0.117)				0.035
IDI	0.196 (0.127–0.264)				<0.001
cfNRI	0.991 (0.513–1.419)				<0.001

Definition of abbreviations: CPB, cardiopulmonary bypass; MRBC, massive transfusion of red blood cells; AUC, area under curve; IDI, integrated discrimination improvement; cfNRI, category-free net reclassification improvement.

a Variable was significant in univariable analyses and initially included as candidate predictors in the stepwise selection procedure, but not selected in the final model.

The classical risk model, constructed without MOTS-c parameters, retained three independent predictors: CPB duration, platelet transfusion volume, and MRBC. In contrast, the modified model incorporated ΔMOTS-c as the sole additional variable (Table 4). Notably, absolute MOTS-c levels at T2 did not exhibit independent predictive value in multivariate analysis. Receiver operating characteristic (ROC) curve analysis demonstrated that ΔMOTS-c alone achieved an area under the curve (AUC) of 0.77 for ARDS prediction (Fig. 1H). The classical model yielded an AUC of 0.824 (95 % CI: 0.754–0.895), whereas the modified model exhibited significantly enhanced discriminative performance, with an AUC of 0.885 (95 % CI: 0.825–0.945). Both models demonstrated adequate calibration via the Hosmer-Lemeshow goodness-of-fit test (Table 4). The Delong test confirmed the statistical superiority of the modified model over the classical model (P = 0.035) (Table 4). Compared to the classical model, the percentage of correct cases reclassified with the modified model was positively elevated (cfNRI = 0.991, 95 % CI: 0.513–1.419, P < 0.001), with a 19.6 % improvement in the integrated discrimination ability (IDI = 0.196, 95 % CI: 0.127–0.264, P < 0.001) (Table 4).

These findings underscore the significant correlation between 24-h serum MOTS-c increments and the development of CPB-induced ARDS. The modified predictive model, integrating MOTS-c dynamics with intraoperative clinical variables, demonstrated superior prognostic utility. The enhanced predictive capacity of ΔMOTS-c may reflect its association with adaptive responses to CPB-induced secondary injury, potentially mediated through antioxidant defense mechanisms. Further investigation is warranted to elucidate the biological pathways underlying this relationship.

### 2.4 MOTS-c attenuates oxidative stress and preserves endothelial barrier integrity in lung ischemia-reperfusion injury

**Fig. 2:**
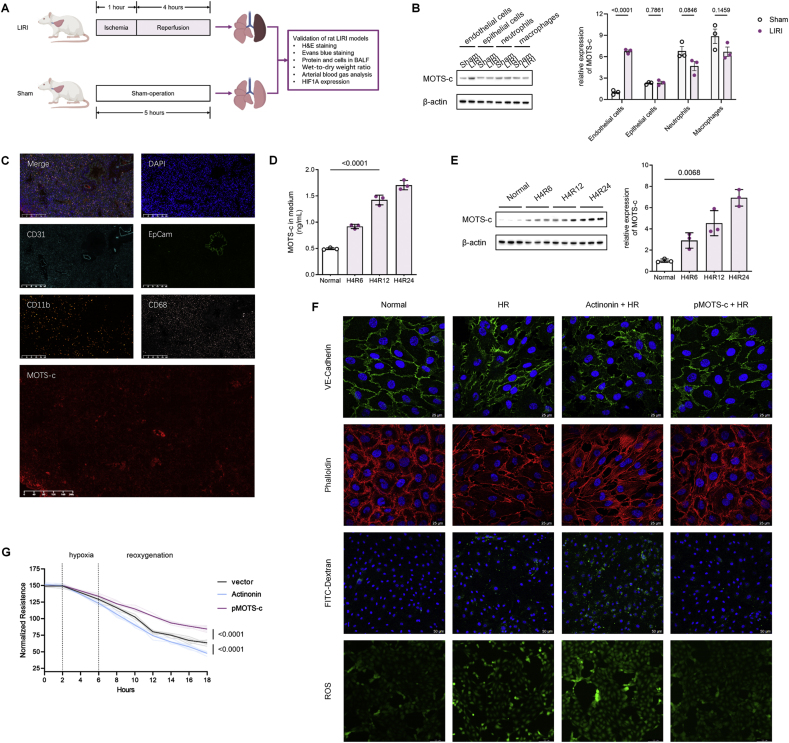

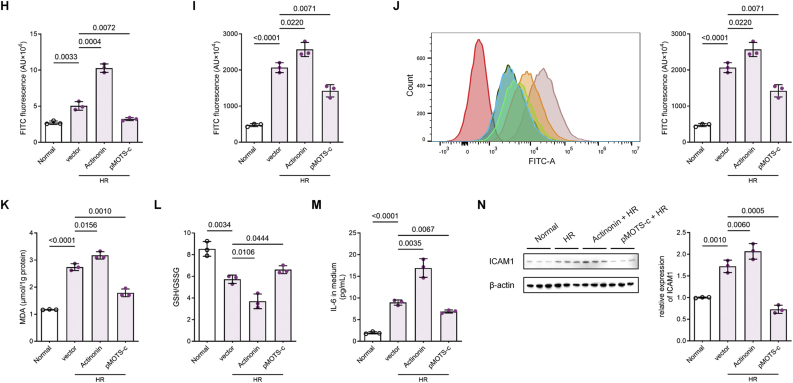
MOTS-c attenuates oxidative stress and preserves endothelial barrier integrity in lung ischemia-reperfusion injury **(A)** Schematic diagram of the rat LIRI model via unilateral pulmonary hilar clamping. **(B)** Western blot analysis of MOTS-c expression levels in sorted endothelial cells, epithelial cells, neutrophils, and macrophages from LIRI and sham rat lungs (n = 3, one-way ANOVA post hoc Student-Newman-Keuls test). **(C)** Immunofluorescence staining of lung tissue sections from LIRI rats, colocalizing MOTS-c with various cell markers. Red, MOTS-c; cyan, CD31; green, EpCAM; orange, CD11b; pink, CD68; blue, DAPI (nuclei). **(D)** Time-dependent increase in extracellular MOTS-c release from HPMVECs during HR (n = 3, one-way ANOVA post hoc Student-Newman-Keuls test). **(E)** Intracellular MOTS-c expression dynamics in HPMVECs during HR (n = 3, one-way ANOVA post hoc Student-Newman-Keuls test). **(F)** Representative immunofluorescence images of VE-cadherin (scale bars: 25 μm), rhodamine-phalloidin (scale bars: 25 μm), FITC-dextran permeability (scale bars: 50 μm), and DCFH-DA ROS staining (scale bars: 100 μm) in HPMVECs under normoxia, hypoxia-reoxygenation (HR), HR with actinonin (150 μM) pretreatment (48 h), and HR with MOTS-c overexpression conditions. **(G)** TEER measurements reflecting barrier function in HPMVECs treated with actinonin, MOTS-c overexpression, or vehicle during HR (n = 6, one-way ANOVA post hoc Student-Newman-Keuls test). **(H–K)** Quantitative analyses of FITC-dextran permeability (H), ROS fluorescence intensity (I), flow cytometry of ROS levels (J), MDA content (K), and GSH/GSSG ratio (L) in HPMVECs under indicated conditions (n = 3, one-way ANOVA post hoc Student-Newman-Keuls test). **(L**–**M)** HR-induced inflammatory activation assessed by IL-6 secretion in cell culture medium (L) and ICAM-1 expression via Western blot (M) (n = 3, one-way ANOVA post hoc Student-Newman-Keuls test). Data are presented as the mean ± SEM. Abbreviations: LIRI, lung ischemia-reperfusion injury; H&E, hematoxylin & eosin; BALF, bronchoalveolar lavage fluid; H4R6, hypoxia for 4 h and reoxygenation for 6 h; H4R12, hypoxia for 4 h and reoxygenation for 12 h; H4R24, hypoxia for 4 h and reoxygenation for 24 h; HR, hypoxia reoxygenation.

**Supplementary Fig. 1:**
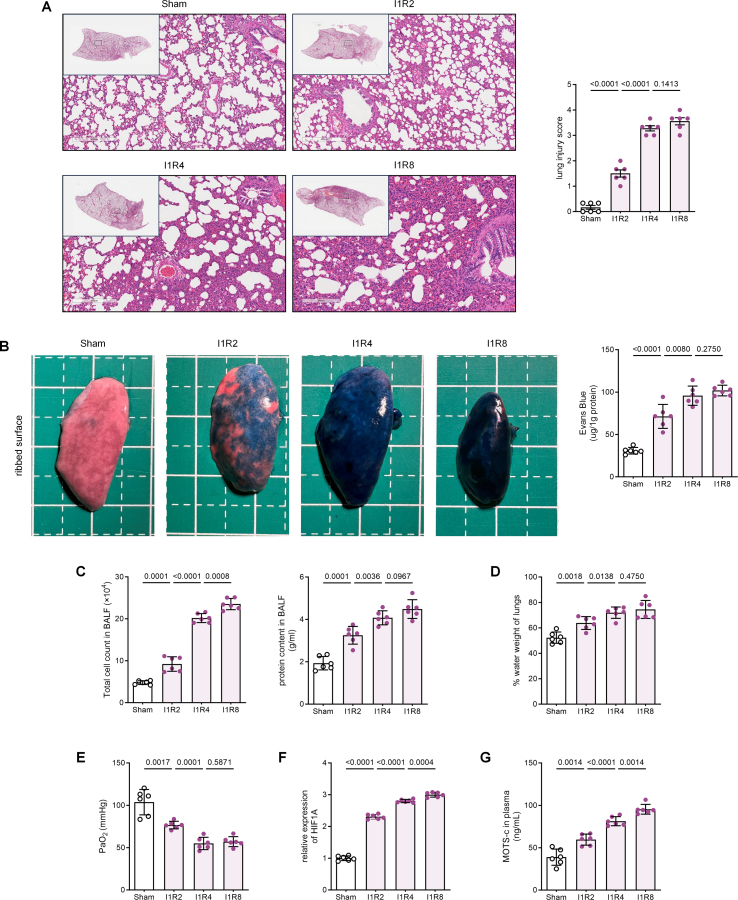
Validation of time-dependent rat LIRI models **(A)** Representative images of H&E-stained lung tissues and lung injury scores (n = 6, one-way ANOVA post hoc Student-Newman-Keuls test). Scale bars: 4 mm. **(B)** Representative images of Evans Blue dye perfusion in sham and LIRI groups (n = 6, one-way ANOVA post hoc Student-Newman-Keuls test). **(C)** Total cell counts and protein concentration in BALF from sham and LIRI groups (n = 6, one-way ANOVA post hoc Student-Newman-Keuls test). **(D)** Lung wet-to-dry weight ratios calculated by measuring water loss during drying of lung tissue (n = 6, one-way ANOVA post hoc Student-Newman-Keuls test). **(E)** PaO_2_ measured via small-animal blood gas analysis (n = 6, one-way ANOVA post hoc Student-Newman-Keuls test). **(F)** RT-qPCR analysis of HIF1A expression in whole-lung tissues (n = 6, one-way ANOVA post hoc Student-Newman-Keuls test). **(H)** Plasma MOTS-c concentrations quantified by ELISA in sham and LIRI rats (n = 6, one-way ANOVA post hoc Student-Newman-Keuls test). Data are presented as the mean ± SEM. Abbreviations: I1R2, ischemia for 1 h and reperfusion for 2 h; I1R4, ischemia for 1 h and reperfusion for 4 h; I1R8, ischemia for 1 h and reperfusion for 8 h; LIRI, lung ischemia-reperfusion injury; BALF, bronchoalveolar lavage fluid; PaO_2_, arterial oxygen tension.

The strong association between circulating MOTS-c levels and CPB-induced ARDS aroused our interest in the role of MOTS-c in the pathophysiology of lung ischemia-reperfusion injury (LIRI). To identify the cellular targets for subsequent mechanistic investigations, we first characterized the distribution and expression dynamics of MOTS-c across major pulmonary cell types during LIRI. A rat LIRI model was established by unilateral pulmonary hilar clamping to recapitulate the pathophysiological features of CPB-induced ARDS (Fig. 2A). A time-gradient LIRI model was established and lung injury was validated through histopathological analysis (Supplementary Fig. 1A–F). Finally, 1-h ischemia and 4-h reperfusion were selected as the model conditions for subsequent formal experiments (Fig. 2A). Cell sorting of lung tissue revealed that endothelial cells exhibited the most pronounced upregulation of MOTS-c expression during LIRI compared to epithelial cells, neutrophils, and macrophages (Fig. 2B). Immunofluorescence staining further confirmed predominant MOTS-c localization in endothelial cells (Fig. 2C). These findings suggest that endothelial MOTS-c may serve as a critical responder to oxidative stress and a protective mediator against LIRI, potentially linked to the heightened vulnerability of endothelial barrier function during LIRI.

**Supplementary Fig. 2:**
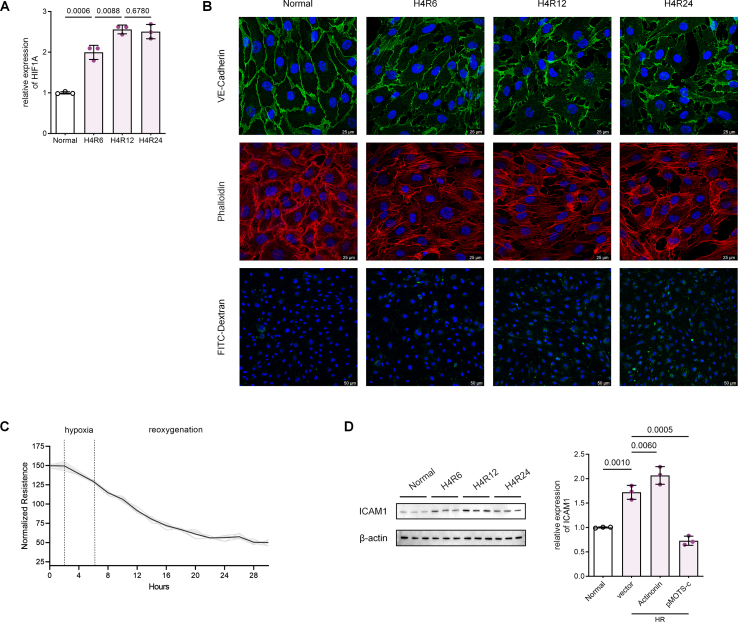
Validation of hypoxia-reoxygenation (HR) time gradients in HPMVECs **(A)** RT-qPCR analysis of HIF1A expression in HR-treated HPMVECs (n = 3, one-way ANOVA post hoc Student-Newman-Keuls test). **(B)** Representative immunofluorescence images of VE-cadherin (scale bars: 25 μm), rhodamine-phalloidin (scale bars: 25 μm), FITC-dextran permeability (scale bars: 50 μm), and DCFH-DA ROS staining (scale bars: 100 μm) in HPMVECs under HR conditions. **(C)** TEER measured every 2 h during 24-h reoxygenation to evaluate endothelial barrier integrity (n = 6). **(D)** Western blot analysis of ICAM1 expression to assess inflammatory activation in HPMVECs under HR gradients (n = 3, one-way ANOVA post hoc Student-Newman-Keuls test). Data are presented as the mean ± SEM. Abbreviations: H4R6, hypoxia for 4 h and reoxygenation for 6 h; H4R12, hypoxia for 4 h and reoxygenation for 12 h; H4R24, hypoxia for 4 h and reoxygenation for 24 h; HR, hypoxia reoxygenation.

**Supplementary Fig. 3:**
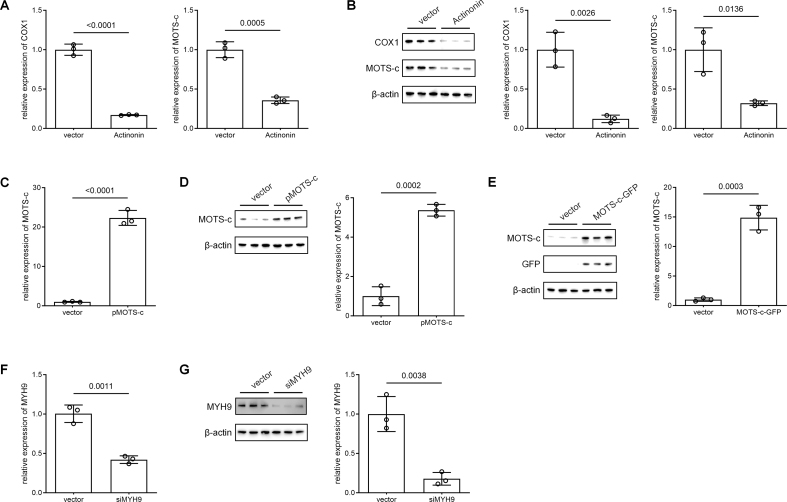
Validation of genetic and pharmacological interventions **(A-B)** Depletion of mitochondrial RNA in HPMVECs treated with actinonin (150 μM, 48 h), confirmed by RT-qPCR and Western blot for COX1 and MOTS-c expression (n = 3, two-tailed *t*-test). **(C**–**D)** Overexpression of MOTS-c in HPMVECs via Lipo3000-mediated transfection of pMOTS-c plasmids, validated by RT-qPCR and Western blot (n = 3, two-tailed *t*-test). **(E)** Western blot detection of GFP-tagged MOTS-c in HPMVECs transduced with lentivirus expressing MOTS-c-GFP fusion protein (n = 3, two-tailed *t*-test). **(F**–**G)** Knockdown of MYH9 in HPMVECs using Lipo3000-transfected siRNA, verified by RT-qPCR and Western blot (n = 3, two-tailed *t*-test). Data are presented as the mean ± SEM.

To validate the functional role of MOTS-c in vitro, primary human pulmonary microvascular endothelial cells (HPMVECs) were subjected to hypoxia-reoxygenation (HR) injury. Cells were cultured in glucose-free medium under hypoxic conditions (1 % O_2_, 4 h) followed by reoxygenation (12 h) to simulate clinical CPB-associated injury timelines. Pilot experiments optimized HR duration based on endothelial barrier dysfunction (Supplementary Fig. 2A–E). Intracellular MOTS-c expression and extracellular release increased progressively during reoxygenation (Fig. 2D and E). To assess MOTS-c's role in barrier regulation, actinonin-mediated mitochondrial RNA depletion (Supplementary Fig. 3A and B) and plasmid-mediated MOTS-c overexpression (Supplementary Fig. 3C and D) were employed. Barrier integrity was evaluated using trans endothelial electrical resistance (TEER), immunofluorescence staining of VE-cadherin and cytoskeleton, and FITC-dextran permeability assays (Fig. 2F–H). MOTS-c depletion exacerbated HR-induced barrier disruption, whereas overexpression mitigated these effects, underscoring MOTS-c's protective role.

Given the important role of oxidative stress in LIRI pathogenesis, we investigated MOTS-c's antioxidant capacity. Reactive oxygen species (ROS) production, quantified via fluorescence microscopy and flow cytometry, was amplified by MOTS-c depletion but suppressed by its overexpression (Fig. 2I and J). Consistently, MOTS-c overexpression reduced malondialdehyde (MDA) levels (a lipid peroxidation marker) and elevated the reduced-to-oxidized glutathione (GSH/GSSG) ratio, while depletion exerted opposing effects (Fig. 2K and L). Furthermore, MOTS-c attenuated HR-induced inflammatory activation, as evidenced by reduced interleukin-6 (IL-6) secretion and intercellular adhesion molecule-1 (ICAM-1) expression (Fig. 2M and N). Collectively, these data demonstrate that MOTS-c mitigates LIRI by counteracting oxidative stress, preserving endothelial barrier integrity, and suppressing inflammation.

### 2.5 MOTS-c undergoes MYH9-Dependent nuclear translocation in response to ROS production

**Fig. 3:**
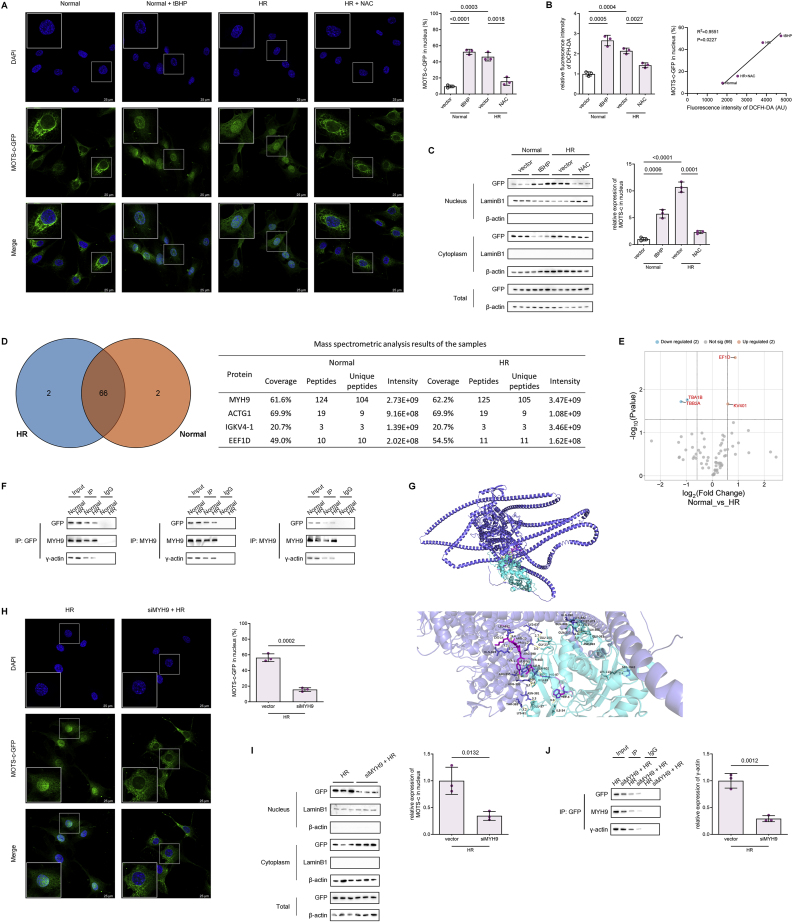
MOTS-c undergoes MYH9-dependent nuclear translocation in response to ROS production **(A)** Representative fluorescence images of HPMVECs stably expressing MOTS-c-GFP under normoxia, hypoxia-reoxygenation (HR), normoxia with tBHP (100 μM), and HR with NAC (10 mM) and quantification of the proportion of MOTS-c-GFP in the nucleus. Green, MOTS-c-GFP; blue, DAPI (nuclei). Scale bars: 20 μm. **(B)** The relative fluorescence intensity of DCFH-DA staining under the same conditions as in (A) and its linear correlation analysis with the proportion of MOTS-c-GFP in the nucleus. **(C)** Western blot analysis of GFP expression in cytoplasmic and nuclear fractions of MOTS-c-GFP HPMVECs under the same conditions as in (A) (n = 3, one-way ANOVA post hoc Student-Newman-Keuls test). **(D)** Liquid chromatography tandem mass spectrometry (LC-MS/MS) analysis of MOTS-c-GFP immunoprecipitates from normoxic and HR-treated HPMVECs, identifying MYH9 and γ-Actin as the top binding partners. **(E)** Volcano plot of IP-MS results labeled with MYH9 and γ-Actin, demonstrating no significant difference between normoxic and HR conditions. **(F)** Western blot validation of MOTS-c-GFP interactions with MYH9 and γ-Actin in immunoprecipitates from normoxic and HR-treated HPMVECs (n = 3). **(G)** Molecular docking simulation using GRAMM, illustrating the interaction between MOTS-c and the MYH9-γ-Actin complex. **(H)** Representative fluorescence images of MOTS-c-GFP HPMVECs transfected with siRNA-MYH9 or siRNA-NC under HR conditions and quantification of the proportion of MOTS-c-GFP in the nucleus, demonstrating reduced nuclear GFP fluorescence in MYH9-depleted cells. Green, MOTS-c-GFP; blue, DAPI (nuclei). Scale bars: 20 μm. **(I)** Western blot analysis of GFP expression in cytoplasmic and nuclear fractions of MOTS-c-GFP HPMVECs transfected with siRNA-MYH9 or siRNA-NC under HR conditions (n = 3, two-tailed *t*-test). **(J)** Western blot analysis of γ-Actin levels in GFP immunoprecipitates from MOTS-c-GFP HPMVECs transfected with siRNA-MYH9 or siRNA-NC, demonstrating reduced γ-Actin binding upon MYH9 depletion (n = 3, two-tailed *t*-test). Data are presented as the mean ± SEM. Abbreviations: HR, hypoxia reoxygenation; tBHP, *tert*-butyl hydroperoxide; NAC, N-acetylcysteine.

In 2018 Kim et al. first reported that MOTS-c dynamically translocates to the nucleus under metabolic stress [13]. Given the hypoxic, serum-deprived, and glucose-deficient microenvironment of pulmonary endothelial cells during LIRI, we hypothesized that reactive oxygen species (ROS) drive MOTS-c nuclear translocation in HR-treated HPMVECs. To visualize dynamics changes of intracellular MOTS-c localization, HPMVECs were transduced with lentivirus to stably express MOTS-c fused with green fluorescent protein (MOTS-c-GFP) (S3E). Fluorescence microscopy revealed predominant cytoplasmic MOTS-c-GFP localization under normoxia, with marked nuclear accumulation following HR (Fig. 3A). Nuclear translocation was ROS-dependent, as *tert*-butyl hydroperoxide (tBHP)-induced ROS under normoxia mimicked HR effects, while the ROS scavenger N-acetylcysteine (NAC) abolished HR-triggered nuclear entry, as further evidenced by the good linear relationship between nuclear MOTS-c fraction and intracellular ROS accumulation (Fig. 3A and B). Subcellular fractionation and GFP-specific western blotting confirmed this redistribution (Fig. 3C).

Previous studies have demonstrated that MOTS-c does not contain the classical nuclear localization sequence (NLS), and its nuclear translocation is highly dependent on the hydrophobic core (_8_YIFY_11_) [13]. This suggests that MOTS-c may rely on its hydrophobic core to bind to other proteins and complete the transport to the nucleus. To identify binding partners, MOTS-c-GFP immunoprecipitation (IP) coupled with mass spectrometry (IP-MS) was performed in normoxic and HR-treated HPMVECs. Liquid chromatography tandem mass spectrometry (LC-MS/MS) revealed that MYH9 and γ-Actin were the most evident binding protein (Fig. 3D). However, the binding strength of either MYH9 or γ-Actin was not statistically different between the two groups (Fig. 3E). MYH9-actin molecular motor is a well-known transport protein complex, and then we demonstrated that they are the key binding proteins for MOTS-c nuclear translocation. IP assay confirmed these interactions in both normoxic and HR conditions (Fig. 3F). Molecular docking simulations using GRAMM further revealed that MOTS-c's hydrophobic core (_8_YIFY_11_) directly engages MYH9-γ-Actin complexes (Fig. 3G), aligning with prior structural predictions.

To substantiate the discovery that MOTS-c underwent nuclear translocation via the MYH9-γ-Actin molecular motor, we disrupted MYH9 expression in MOTS-c-GFP HPMVEC with siRNA-MYH9 transfection (Supplementary Fig. 3F and G). Compared with the scrambled knockdown control, dramatically decreased green fluorescence and GFP expression in the nucleus were observed in MYH9 knockdown cells (Fig. 3H and I). Repeated IP revealed that γ-Actin in the pull-down proteins decreased with MYH9 depletion (Fig. 3J), demonstrating that MYH9-γ-Actin complexes are essential for MOTS-c transport.

### 2.6 CK2-dependent MYH9 Ser1943 Phosphorylation Governs MOTS-c Nuclear Translocation and Endothelial Protection

**Fig. 4:**
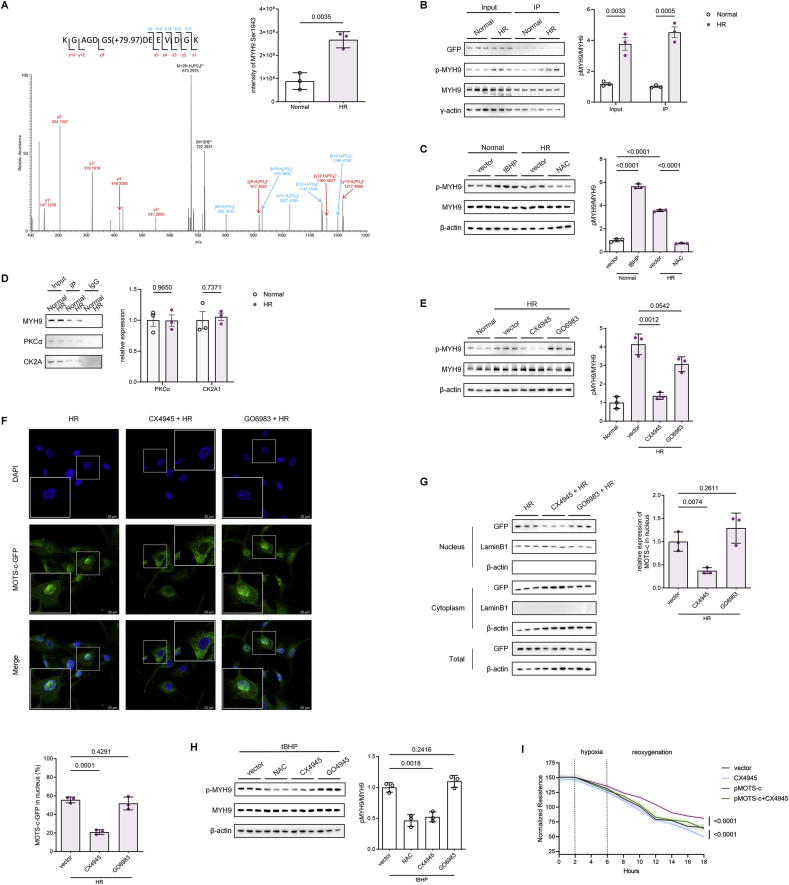

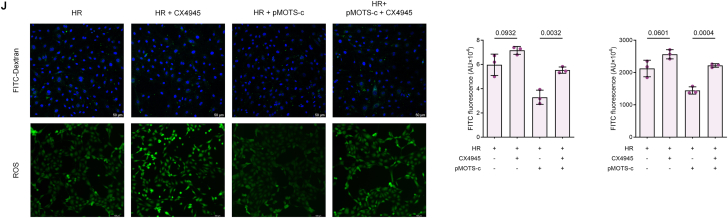
CK2A-Mediated MYH9 Ser1943 Phosphorylation Governs MOTS-c Nuclear Translocation and Endothelial Protection **(A)***Left*: Higher-energy collision-induced dissociation (HCD) MS/MS spectrum of the human MYH9 phosphopeptide KGAGDGSDEEVDGK ([M+2H]^2+^ ion at *m*/*z* 722.2874). Predicted b- and y-ions are annotated. *Right*: MYH9 Ser1943 phosphorylation intensity in normoxia versus HR groups (n = 3, two-tailed *t*-test). **(B)** Immunoprecipitation of MOTS-c-GFP with MYH9 in HPMVECs under normoxia or HR, followed by Western blot analysis of p-MYH9/t-MYH9 ratios (n = 3, two-tailed *t*-test). **(C)** Western blot analysis of MYH9 Ser1943 phosphorylation in HPMVECs treated with tBHP (100 μM) under normoxia or NAC (10 mM) during HR (n = 3, one-way ANOVA post hoc Student-Newman-Keuls test). **(D)** Immunoprecipitation of MYH9 with CK2A and PKCα in MOTS-c-GFP HPMVECs under normoxia or HR (n = 3, two-tailed *t*-test). **(E)** Western blot analysis of MYH9 phosphorylation in HPMVECs pretreated with CK2A inhibitor CX4945 (10 μM, 2 h) or PKCα inhibitor GO6983 (100 nM, 2 h) prior to HR (n = 3, one-way ANOVA post hoc Student-Newman-Keuls test). **(F)** Representative fluorescence imaging of MOTS-c-GFP nuclear translocation in HPMVECs pretreated with CX4945 (10 μM, 2 h) or GO6983 (100 nM, 2 h) prior to HR and quantification of the proportion of MOTS-c-GFP in the nucleus. Green, MOTS-c-GFP; blue, DAPI (nuclei). Scale bars: 25 μm. **(G)** Subcellular fractionation and Western blot analysis of GFP localization in cytoplasmic and nuclear compartments (n = 3, one-way ANOVA post hoc Student-Newman-Keuls test). **(H)** Western blot analysis of MYH9 phosphorylation in HPMVECs pretreated with NAC (10 mM), CX4945 (10 μM), or GO6983 (100 nM) for 2 h, followed by tBHP (100 μM) stimulation for 16 h (n = 3, one-way ANOVA post hoc Student-Newman-Keuls test). **(I)** TEER measurements in MOTS-c-overexpressing HPMVECs pretreated with CX4945 (10 μM, 2 h) during HR (n = 6, two-tailed *t*-test). **(J)** Representative immunofluorescence images of FITC-dextran permeability (scale bars: 50 μm) and DCFH-DA ROS staining (scale bars: 100 μm) in HPMVECs pretreated with CX4945 (10 μM, 2 h) during HR (n = 3, one-way ANOVA post hoc Student-Newman-Keuls test). Data are presented as the mean ± SEM. Abbreviations: HR, hypoxia reoxygenation; IP, immunoprecipitation; tBHP, *tert*-butyl hydroperoxide; NAC, N-acetylcysteine.

Reanalysis of IP-MS data revealed no significant difference in MOTS-c-GFP binding to MYH9 under HR versus normoxia. However, phosphorylation profiling using a post-translational modification database identified MYH9 phosphorylation at Ser1943—a residue within the C-terminal rod domain critical for cargo binding—as the sole modification significantly upregulated during HR (Fig. 4A). Ser1943 of MYH9 is located in the C-terminal rod domain and nonhelical tailpiece, which is critical for regulating filament formation and cargo binding, as reported by Ricketson D et al., in 2010. Immunoprecipitation with GFP antibodies confirmed elevated phosphorylated-to-total MYH9 (p-MYH9/t-MYH9) ratios in HR-treated cell lysates and IP eluates (Fig. 4B). Pharmacological modulation further validated ROS dependence, as tBHP-induced ROS under normoxia mimicked HR-driven MYH9 phosphorylation, while the ROS scavenger NAC suppressed this modification during HR (Fig. 4C). These findings establish MYH9 Ser1943 phosphorylation as a redox-sensitive prerequisite for MOTS-c nuclear translocation.

CK2A and PKCα are known to be common MYH9 ser1943 phosphorylases. To identify the kinase responsible for MYH9 phosphorylation, IP of MYH9 in HR-treated cells detected co-precipitated CK2A and PKCα (Fig. 4D). Selective kinase inhibition demonstrated that CK2A inhibitor CX4945, but not PKCα inhibitor GO6983, abolished MYH9 Ser1943 phosphorylation (Fig. 4E). Western blot and fluorescence microscopy also confirmed that the nuclear translocation of MOTS-c was inhibited after CX4945 but not GO6983 treatment (Fig. 4F and G). These results unequivocally identify CK2A as the dominant kinase regulating MYH9 phosphorylation and MOTS-c transport.

To assess the functional relevance of MYH9 phosphorylation, endothelial barrier integrity was evaluated in MOTS-c-overexpressing HPMVECs pretreated with CX4945. As shown in TEER and ROS assay, CX4945 pretreatment aggravated the hypoxia-reoxygenation induced barrier disruption and ROS accumulation, effectively negating the protective effects of MOTS-c (Fig. 4I and J). Immunofluorescence revealed enlarged intercellular gaps and cytoskeletal disorganization in CX4945-treated cells, confirming that MOTS-c's barrier preservation requires CK2A-dependent MYH9 phosphorylation and subsequent nuclear translocation. Collectively, these findings delineate a ROS-CK2A-MYH9 signaling axis that coordinates MOTS-c nuclear translocation, positioning this pathway as a central mechanistic link between oxidative stress, mitochondrial-nuclear communication, and endothelial barrier protection in ischemia-reperfusion injury.

### 2.7 Nuclear MOTS-c Exhibits Transcriptional Factor-like activity by Directly Binding to Antioxidant Gene Promoters

**Fig. 5:**
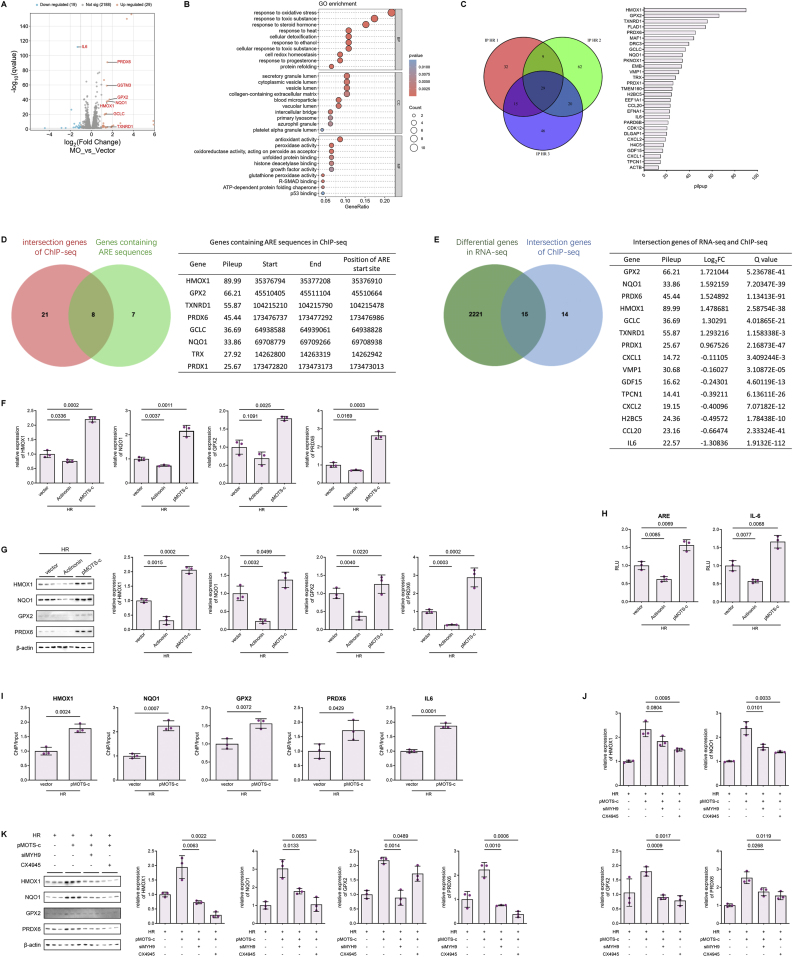
Nuclear MOTS-c Exhibits Transcriptional Factor-like Activity by Directly Binding to Antioxidant Gene Promoters **(A)** Volcano plot of RNA-seq data from MOTS-c-overexpressing HPMVECs subjected to hypoxia-reoxygenation (HR). Differentially expressed genes were filtered (|fold change| >2, Q-value <0.05), with key antioxidant enzymes and pro-inflammatory factors highlighted in red. **(B)** Gene Ontology (GO) enrichment analysis of differentially expressed genes, categorized into biological processes (BP), cellular components (CC), and molecular functions (MF). **(C)***Left*: Venn diagram of genes identified in three independent ChIP-seq experiments. *Right*: Peaks corresponding to overlapping genes. **(D)***Left*: Intersection of ChIP-seq-derived genes with promoters containing AREs. *Right*: Genomic coordinates of overlapping genes. **(E)** Intersection of RNA-seq and ChIP-seq datasets and core analysis results. **(F**–**G)** RT-qPCR and Western blot validation of HMOX1, NQO1, GPX2, and PRDX6 expression in HPMVECs with MOTS-c modulation under HR (n = 3, one-way ANOVA post hoc Student-Newman-Keuls test). (H) Luciferase reporter gene assays confirming transcription factor-like effects of MOTS-c on the AREs of antioxidant genes and the promoter regions of IL6 (n = 3, one-way ANOVA post hoc Student-Newman-Keuls test). **(I)** ChIP-qPCR confirming MOTS-c binding to promoter regions of HMOX1, NQO1, GPX2, and PRDX6 in MOTS-c-overexpressing HPMVECs post-HR (n = 3, two-tailed *t*-test). **(J**–**K)** RT-qPCR and Western blot analysis of HMOX1, NQO1, GPX2, and PRDX6 expression in MOTS-c-overexpressing HPMVECs pretreated with MYH9 siRNA or CK2A inhibitor CX4945 (10 μM, 2 h) under HR (n = 3, one-way ANOVA post hoc Student-Newman-Keuls test). Data are presented as the mean ± SEM. Abbreviations: HR, hypoxia reoxygenation; IP, immunoprecipitation; ChIP-seq, chromatin immunoprecipitation sequencing; ARE, antioxidant response element; RNA-seq, RNA sequencing; RLU, relative light unit.

To investigate the biological activity of MOTS-c following ROS-CK2A-MYH9-mediated nuclear translocation, RNA sequencing (RNA-seq) was performed in HPMVECs overexpressing MOTS-c via pMOTS-c plasmid transfection and subjected to hypoxia-reoxygenation (HR). Differential gene expression analysis (absolute fold change >2, Q-value <0.05) identified 29 upregulated and 19 downregulated genes (Fig. 5A). Upregulated genes included well-characterized antioxidant genes such as PRDX6, GSTM3, GPX2, NQO1, HMOX1, GCLC, and TXNRD1, while pro-inflammatory mediators like IL6 and CXCL2 were significantly downregulated. Gene Ontology (GO) enrichment analysis revealed that biological processes (BP) were predominantly enriched in “oxidative stress response”, “response to toxic substances”, and “steroid hormone response”, whereas molecular functions (MF) included “antioxidant activity”, “peroxidase activity”, and “oxidoreductase activity acting on peroxides” (Fig. 5B). These findings robustly demonstrate that MOTS-c's biological functions converge on antioxidant defense and anti-inflammatory regulation, corroborating its phenotypic protective effects observed in prior experiments.

To identify MOTS-c's direct genomic targets, chromatin immunoprecipitation sequencing (ChIP-seq) was conducted in HPMVECs stably expressing MOTS-c-GFP using ChIP-grade GFP antibodies. Three independent ChIP-seq experiments were performed, and intersection analysis of the results yielded 29 high-confidence MOTS-c-bound genes (Fig. 5C). Cross-referencing these genes with known antioxidant response element (ARE)-containing promoters identified 8 overlapping genes (HMOX1, NQO1, etc.) (Fig. 5D). Integration of RNA-seq and ChIP-seq datasets further narrowed the list to 15 genes whose promoters were directly bound by MOTS-c and transcriptionally regulated (Fig. 5E). These included ARE-driven antioxidant genes (GPX2, NQO1, HMOX1, PRDX6, GCLC, TXNRD1, PRDX1) and inflammation-related genes (IL6, CCL20, CXCL2, CXCL1), demonstrating MOTS-c's dual role as a transcriptional activator of antioxidant pathways and a repressor of pro-inflammatory signals.

The transcriptional regulatory role of nuclear MOTS-c was experimentally validated using RT-qPCR and Western blotting. HR increased the expression of HMOX1, NQO1, GPX2, and PRDX6, which was further amplified by MOTS-c overexpression (Fig. 5F and G). Luciferase reporter assays confirmed MOTS-c's direct binding to ARE-containing promoters and the IL6 promoter (Fig. 5H). ChIP-qPCR validated MOTS-c's occupancy at these loci (Fig. 5I). Critically, silencing MYH9 or inhibiting CK2A-mediated phosphorylation abolished MOTS-c-driven transcriptional activation (Fig. 5J and K), unequivocally establishing that nuclear translocation via the ROS-CK2A-MYH9 axis is indispensable for its transcriptional activity.

### 2.8 Exogenous Administration of Synthetic MOTS-c confers lung protection in rat LIRI models

**Fig. 6:**
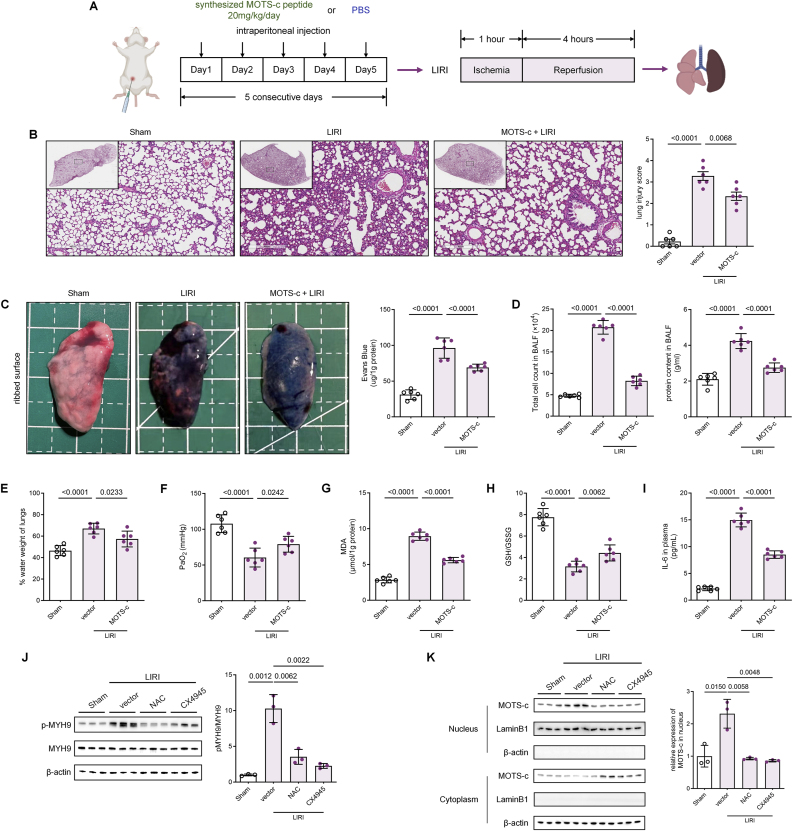

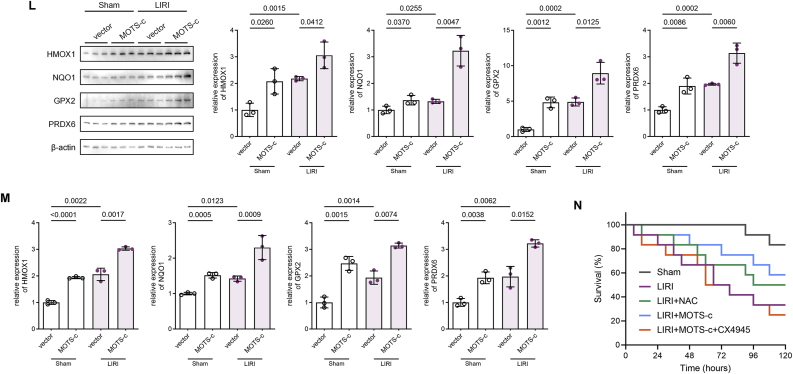
Exogenous Administration of Synthetic MOTS-c Exerts Lung Protection in Rat LIRI Models Through Conserved Mechanisms **(A)** Schematic diagram of the rat LIRI model after 5 consecutive days of intraperitoneal administration of synthesized MOTS-c peptide (20 mg/kg). **(B)** Representative images of H&E-stained lung tissues and lung injury scores in sham, LIRI-only, and MOTS-c-pretreated LIRI groups (n = 6, one-way ANOVA post hoc Student-Newman-Keuls test). Scale bars: 4 mm. **(C)** Representative images of Evans blue dye perfusion in sham, LIRI-only, and MOTS-c-pretreated LIRI groups (n = 6, one-way ANOVA post hoc Student-Newman-Keuls test). **(D)** Total cell counts and protein concentration in BALF from sham, LIRI-only, and MOTS-c-pretreated LIRI groups (n = 6, one-way ANOVA post hoc Student-Newman-Keuls test). **(E)** Lung wet-to-dry weight ratios calculated by measuring water loss during drying of lung tissue among sham, LIRI-only, and MOTS-c-pretreated LIRI groups (n = 6, one-way ANOVA post hoc Student-Newman-Keuls test). **(F)** PaO_2_ measured via small-animal blood gas analysis in sham, LIRI-only, and MOTS-c-pretreated LIRI groups (n = 6, one-way ANOVA post hoc Student-Newman-Keuls test). **(G**–**H)** MDA concentrations and GSH/GSSG ratios in whole-lung lysates of sham, LIRI-only, and MOTS-c-pretreated LIRI groups (n = 6, one-way ANOVA post hoc Student-Newman-Keuls test). **(I)** Plasma IL-6 concentrations in sham, LIRI-only, and MOTS-c-pretreated LIRI groups (n = 6, one-way ANOVA post hoc Student-Newman-Keuls test). **(J)** Phosphorylated MYH9 to total MYH9 ratio in whole-lung tissues of sham, LIRI-only, and LIRI groups pretreated with CX4945 (75 mg/kg, oral gavage) or N-acetylcysteine (NAC, 300 mg/kg, intraperitoneal injection) 24 h prior to modeling (n = 3, one-way ANOVA post hoc Student-Newman-Keuls test). **(K)** Subcellular distribution of MOTS-c in cytoplasmic and nuclear fractions of whole-lung tissue from sham, LIRI-only, CX4945-pretreated LIRI, and NAC-pretreated LIRI groups (n = 3, one-way ANOVA post hoc Student-Newman-Keuls test). **(L**–**M)** Western blot and RT-qPCR analyses of HMOX1, NQO1, GPX2, and PRDX6 expression in whole-lung tissues of sham and LIRI groups pretreated with synthetic MOTS-c (20 mg/kg, intraperitoneal injection) or solvent for 5 consecutive days (n = 3, one-way ANOVA post hoc Student-Newman-Keuls test). **(N)** 5-day survival curves of sham, LIRI-only, NAC-pretreated LIRI, MOTS-c-pretreated LIRI, and MOTS-c + CX4945-pretreated LIRI groups (n = 12, log-rank test). Data are presented as the mean ± SEM. Abbreviations: LIRI, lung ischemia-reperfusion injury; BALF, bronchoalveolar lavage fluid; PaO_2_, arterial oxygen tension; MDA, malondialdehyde; GSH, reduced glutathione; GSSG, oxidized glutathione; NAC, N-acetylcysteine.

Preventive strategies targeting MOTS-c for mitigating CPB-induced ARDS show promise, but the mitochondria-encoded nature of MOTS-c complicates investigations into its upstream regulatory mechanisms. To facilitate clinical translation, we investigated whether intraperitoneal administration of synthetic MOTS-c recapitulates the protective effects of endogenous MOTS-c. The rat airway clamp model of LIRI was established following intraperitoneal administration of synthesized MOTS-c peptide at a dose of 20 mg/kg/day for five consecutive days (Fig. 6A). This protocol ensured sufficient exogenous MOTS-c reserves in the rats prior to model construction, regardless of the inherent capacity for endogenous MOTS-c expression mobilization. We assessed pathologic improvements via hematoxylin and eosin (H&E) staining, which revealed attenuated lung injury in MOTS-c-treated groups compared to untreated LIRI models, corroborated by Evans blue assay results (Fig. 6B and C). Furthermore, MOTS-c administration significantly reduced lung water content, BALF protein levels and cellular infiltration, indicating preservation of the air-blood barrier (Fig. 6D and E). Improved oxygenation capacity was evidenced by elevated arterial oxygen tension (PaO_2_) in MOTS-c-treated rats post-LIRI (Fig. 6F). Serum ELISA and biochemical analyses demonstrated that MOTS-c treatment markedly decreased plasma IL-6, lung tissue MDA, and lung tissue GSSG/GSH ratio, consistent with attenuated inflammation and oxidative stress (Fig. 6G–I). Survival analysis further revealed that MOTS-c pretreatment significantly enhanced 5-day survival rates in rats with severe lung injury, and even demonstrated superiority over the NAC-pretreated group (Fig. 6N). Collectively, these findings indicate that exogenous synthetic MOTS-c mitigates LIRI-induced deterioration of lung barrier function and oxygenation by suppressing inflammatory and oxidative pathways.

To determine whether the lung-protective effect of exogenous MOTS-c depend on antioxidant gene regulation via nuclear translocation, we first examined phosphorylation of MYH9,a prerequisite for MOTS-c nuclear translocation mediated by CK2A. In rat lung tissues, phosphorylation of MYH9 at Ser1943 was significantly upregulated post-LIRI compared to sham controls, which could be inhibited by pretreatment with the ROS inhibitor NAC or the CK2A inhibitor CX4943 (Fig. 6J). Then pre-administered MOTS-c in all groups was conducted to examine its intracellular distribution under different conditions. Consistent with the changes in phosphorylation of MYH9, MOTS-c translocated to the nucleus following rat LIRI modeling, and pre-treatment with NAC and CX4945 effectively blocked this process (Fig. 6K), confirming that exogenous MOTS-c undergoes nuclear translocation in response to LIRI mediated by ROS-CK2A-MYH9 axis. Finally, RT-qPCR and Western Blot revealed that MOTS-c upregulated the expression of antioxidant genes HMOX1, NQO1, GPX2, and PXDR6 at transcriptional and translational levels (Fig. 6L and M). Survival analysis similarly confirmed that CX4945 pretreatment reversed the 5-day survival improvement associated with MOTS-c administration (Fig. 6N). In summary, our rat LIRI model demonstrated that exogenous MOTS-c administration successfully recapitulated the biological processes mediated by endogenous MOTS-c in vitro. These findings strongly suggest that pharmacological strategies targeting MOTS-c signaling pathways hold significant preventive potential for mitigating lung injury.

## 3 Conclusion

MOTS-c attenuates CPB-induced lung injury through ROS-CK2A-MYH9-mediated nuclear translocation, where it exerts transcription factor-like activity to activate antioxidant defenses and suppress inflammation. Clinically, its dynamic post-CPB increment serves as a prognostic indicator for ARDS, while exogenous MOTS-c recapitulates protection through analogous molecular pathways, positioning MOTS-c as a promising biomarker and preventive strategy.

## 4 Discussion

According to the endosymbiosis hypothesis, mitochondria originated in prokaryote, and it is likely that this endosymbiotic promitochondrial bacteria used peptides encoded in their genomes to communicate with our ancestral cells, a communication system still used by bacteria [34]. It is plausible that the two genomes coevolved to regulate each other to coordinate cellular functions. Mitochondrial to nucleus (retrograde) communication mechanisms in response to cellular stress, including mtUPR [35] and damage-associated molecular patterns (DAMPs) [36], have been well described but are known to be mediated by nuclear encoded proteins/peptides. Mitochondria-derived peptide, which is encoded by small open reading frames of mitochondrial genome, includes Humanin, MOTS-c, SHLP1-6. The unique genetic system of this bacterial origin has long been poorly understood. In 2011 *Cell* revealed the high complexity of transcriptional regulation of the mitochondrial genome, and many mysteries remain to this day [37]. This also leads to the fact that there are few in-depth studies on the upstream regulatory pathways and downstream regulatory mechanisms of MOTS-c since it was first characterized in 2015 [17]. In 2018, Lee et al. found that MOTS-c can undergo nuclear translocation under metabolic stress conditions, and this process is highly sequence dependent but not dose dependent. Although MOTS-c does not have the classical NLS sequence, researchers found that the _8_YIFY_11_ sequence is essential for the nuclear translocation of MOTS-c [25]. This breakthrough deeply inspired us that MOTS-c may rely on the hydrophobic core _8_YIFY_11_ to cooperate with other proteins for nuclear translocation. Our findings unveil MOTS-c as a critical mediator of antioxidant defense and endothelial protection during CPB-induced lung injury, operating through a ROS-CK2A-MYH9 axis that orchestrates its nuclear translocation and transcriptional activity. This research represents the first comprehensive exploration of MOTS-c's nuclear translocation characteristics and mitochondrial-nuclear communication mechanisms since their initial discovery in 2018, not only expanding the mechanistic understanding of mitochondrial retrograde signaling but also underscoring the translational potential of MOTS-c as both a biomarker and preventive agent.

Central to this study is the discovery that MOTS-c nuclear translocation is mechanistically coupled to oxidative stress via MYH9 phosphorylation. While prior work identified MOTS-c as a metabolic regulator, our data reveal its role in bridging mitochondrial dysfunction to nuclear antioxidant responses. The dependency on CK2A-mediated MYH9 Ser1943 phosphorylation suggests a redox-sensitive “molecular switch” that licenses MOTS-c transport, similar to cargo-mediated MYH7 motor activation [38]. This finding aligns with emerging paradigms of myosin motors as dynamic regulators of subcellular trafficking under stress, yet diverges from classical nuclear transport mechanisms reliant on canonical NLS sequences [39]. The reliance on MYH9-γ-Actin complexes raises intriguing questions about the evolutionary conservation of this pathway and its potential involvement in other oxidative stress-related pathologies, such as sepsis or acute kidney injury, where endothelial barrier dysfunction is prominent [40,41]. The important role of MYH9-γ-actin in MOTS-c nuclear translocation inspired us that this process likely integrates cytoskeletal mechanics and nuclear envelope plasticity [42]. Future studies should clarify whether MOTS-c leverages physiological nuclear restructuring (e.g., mitotic NPC disassembly) for entry and how lamin isoforms influence tissue-specific trafficking, bridging mitochondrial signaling with nuclear biomechanics.

Clinically, the dynamic rise in serum MOTS-c (ΔMOTS-c) within 24 h post-CPB emerged as a superior predictor of ARDS compared to static measurements. This temporal association implies that MOTS-c release reflects an adaptive response to ongoing reperfusion injury, with diminished increments signaling inadequate antioxidant reserve. The dissociation between ΔMOTS-c and intraoperative variables (e.g., CPB duration) further suggests that individual variability in MOTS-c induction—potentially influenced by genetic or epigenetic factors—may determine susceptibility to postoperative complications. This hypothesis is bolstered by the in vitro correlation between MOTS-c expression and injury severity, positioning ΔMOTS-c as a biomarker of intrinsic stress resilience. Future studies could explore genetic polymorphisms in mitochondrial genes or nuclear-encoded transporters (e.g., MYH9) that modulate MOTS-c dynamics.

Preventive administration of synthetic MOTS-c in rodent models recapitulated endogenous protective effects, attenuating oxidative damage and improving survival. Unfortunately, numerous interventions that show promise in ALI models have failed in clinical trials. For example, although corticosteroids or NAC may theoretically reduce inflammation and oxidative stress, their beneficial effects on mortality have not been demonstrated in subsequent clinical studies [43,44]. Therefore, although the biological process of MOTS-c nuclear translocation is definite, the experimental results in animals need to be treated with caution, and more pharmacological experiments need to be improved.

In conclusion, this study delineates a novel axis of mitochondrial-nuclear communication wherein MOTS-c serves as both a sentinel of oxidative stress and an effector of antioxidant transcription. The dual utility of MOTS-c—as a dynamic biomarker and a preventive peptide—positions it at the forefront of personalized approaches to post-CPB care. Future investigations should prioritize elucidating genetic determinants of MOTS-c responsiveness, refining delivery strategies for clinical use, and exploring its role in broader contexts of ischemia-reperfusion injury.

### 4.1 Limitation

Several limitations warrant consideration. First, the exclusive focus on endothelial cells, while justified by their barrier vulnerability, leaves open whether MOTS-c exerts similar roles in alveolar epithelial or immune cells. Organ-on-chip models co-culturing multiple cell types could elucidate cell-specific contributions to lung injury and repair. Second, the clinical cohort, though prospectively designed, was limited in sample size and single-center recruitment, necessitating validation in larger, multicenter trials. Finally, while our work establishes MYH9 phosphorylation as a key regulatory step and demonstrates ROS-dependent CK2A activation during hypoxia-reoxygenation, the upstream mechanisms linking ROS to CK2A warrant deeper exploration. Potential candidates include redox-sensitive kinases or mitochondrial-derived signals that amplify CK2A activation under oxidative stress. Notably, emerging evidence highlights a bidirectional regulatory relationship between MOTS-c and CK2. A recent study revealed that MOTS-c directly binds to and activates CK2 in cell-free systems, positioning CK2 as both a mediator and a functional target of MOTS-c signaling. This interaction exhibits tissue-specific modulation: systemic MOTS-c administration enhances CK2 activity in skeletal muscle, promoting glucose uptake and mitigating atrophy, while suppressing CK2 in adipose tissue through differential regulation of CK2-interacting proteins. Within the framework of our study, the ROS-CK2A-MYH9 axis may represent a nexus where mitochondrial retrograde signaling (via MOTS-c) intersects with kinase-driven cytoskeletal remodeling to orchestrate adaptive responses. Future investigations should explore how tissue-specific CK2 interactomes modulate MOTS-c's transcriptional activity in pulmonary endothelium versus other organs, potentially informing targeted delivery strategies to optimize its preventive efficacy.

## 5 Methods

1.Human study design and ethics

The prospective, observational, single-center, controlled trial was conducted at the First Affiliated Hospital with Nanjing Medical University (Nanjing, China) from January 2023 to December 2023, in accordance with the Declaration of Helsinki. The study protocol was approved by the Institutional Review Board (2023-SR-075), and comprehensive written informed consent was obtained from all participants. Patients underwent comprehensive preoperative assessments, including medical history review, physical examination, and laboratory analyses. The primary objective was to investigate the association between circulating MOTS-c levels and the development of CPB-induced ARDS. Secondary aims included evaluating predictive efficacy of serum MOTS-c levels for ARDS.

2.Participants and sample collection

Eligibility screening was performed for all patients undergoing cardiac surgery with CPB during the study period. Exclusion criteria included off-pump surgery, preexisting lung injury (e.g., chronic obstructive pulmonary disease, pleural effusions or atelectasis), prior cardiac surgery, congenital heart disease, acute heart failure, inflammatory or autoimmune diseases, infectious or malignant tumors, transplant recipients, immunosuppressive therapy (e.g., corticosteroids), or investigator affiliation. All participants were provided with routine care, adhering to current guidelines. Clinical data were extracted from medical records, encompassing demographics (age, sex, BMI), medical history, intraoperative variables, and postoperative outcomes.

4 mL arterial blood samples were collected from the patients' arterial catheters before skin incision after anesthesia (T0), after returning to the intensive care unit after CPB surgery (T1), and 24 h after CPB surgery (T2). The collected blood samples were immediately centrifuged in the core laboratory to obtain serum, packaged and stored at −80 °C. After the enrollment and blood sample collection of all patients, the blood samples were thawed and underwent immediate analysis for MOTS-c concentration using the commercially available ELISA kits (EIAab, #E15563h, China). Each sample was assayed in duplicate for accuracy and reliability.

3.Rat LIRI models

Animal experiments in this study were conducted in accordance with the Care and Use of Laboratory Animal guidelines of the Animal Care Facility of Nanjing Medical University (China), and approved by the Institutional Animal Care and Use Committee (IACUC-2105010). Male Sprague-Dawley rats (8–10 weeks old, 200–250 g) were randomly grouped according to experimental needs. Anesthesia was induced using ketamine hydrochloride (100 mg/kg) and xylazine (10 mg/kg). Following intubation with a 22-gauge catheter, mechanical ventilation was maintained using a HUAYON ventilator (Shenzhen, China). A left thoracotomy was performed to occlude the left pulmonary hilum with a non-invasive vascular clamp for 60 min, followed by reperfusion.

4.Rat lung histopathological and functional assessments

Lung tissues were fixed in 4 % paraformaldehyde for 48 h, embedded in dehydrated paraffin, and sectioned into 4 μm slices. The sections were rehydrated in an alcohol gradient and stained with hematoxylin and eosin (H&E). Lung injury scores were evaluated and averaged by three blinded pathologists based on four criteria, including neutrophil infiltration, alveolar edema, alveolar hemorrhage, and lung parenchymal abnormalities. Lung injury was rated as 0 to 4 points based on its severity: 0, no injury; 1, less than 25 % injury; 2, 25–50 % injury; 3, 50–75 % injury; and 4, almost 100 % injury.

After the model was established, the right bronchus was clipped, and 1 mL sterile Hanks balanced saline buffer was pushed into the left lung of the model through the tracheal cannula. After repeated lavage for three times, the lavage fluid was extracted and centrifuged at 3000r/min for 15min at 4 °C, and the supernatant was bronchoalveolar lavage fluid (BALF). Total protein concentration in BALF was determined using the BCA Protein Assay kit (Beyotime, #P0010, China). Inflammatory cell counts in BALF were determined via hemocytometer.

Lung vascular permeability was assessed via Evans blue dye extravasation. Briefly, after LIRI modeling, a 1 % Evans Blue solution (NO. MB4680-1, Meilunbio) was administered intravenously through the tail vein at a dosage of 30 mg/kg at the onset of reperfusion. After reperfusion, the left ventricle of the heart was perfused with PBS (pH 7.4) until the effluent was colorless and clear, thereby eliminating free Evans Blue from the bloodstream. The left lung tissue was then removed and carefully blotted to eliminate surface moisture. The excised lung tissue underwent drying at 72 °C for a duration of 24 h, after which its dry weight was measured. The dried tissue was subsequently immersed in 1 mL of formamide (MCE, #HY-Y0842, USA) and incubated at 37 °C for 24 h to facilitate the extraction of the Evans Blue dye. Following this, the absorbance was quantified at 620 nm using a spectrophotometer.

Arterial partial pressure of oxygen (PaO_2_) was measured at the end of modeling as a necessary index to reflect oxygenation function. Arterial blood samples were collected via the left femoral artery with a 1 mL heparin-rinsed syringe, and arterial blood gas analysis was performed immediately using PL2000PLUS Blood Gas Biochemistry Analyzer (PERLONG MEDICAL, China).

5.Cell sorting from rat lung tissue

Epithelial cells, endothelial cells, and macrophages in lung tissue were sorted using magnetic beads labeled with corresponding antibodies. The brief steps for magnetic bead sorting are as follows. The left lung was rinsed via pulmonary artery catheterization after LIRI modeling or sham operation. The lungs were resected en bloc and moved into the clean bench, washed twice with normal saline, and injected 6 mL protease solution (containing 10 U/ml Dispase, 10 % fetal bovine serum (FBS), 10 U/ml DNase I solubilized in 1640 Medium) through the trachea. Then transfer the lungs into a 50-mL centrifugal tube containing 10 mL protease solution to digest lung tissues completely at 37 °C for 45 min; afterwards cut the lung lobes into small pieces by ophthalmic scissors and filter the digested tissue through a 75 μm filter and then a 40 μm filter; finally, centrifugate the samples at a speed of 300 g for 5 min. Precipitates were isolated single cells of lung tissue, which were then sorted by magnetic beads using different antibodies. First, remove the supernatant and resuspend cell precipitation in dispersing agent (containing 10 % FBS, 10 U/mL DNase I solubilized in RPMI 1640 medium), divided into three equal volumes for subsequent simultaneous sorting of the three cell types. 1 μg anti-rat antibodies were separately mixed with the suspension, and then incubate at 4 °C for 15 min. Second, wash the suspension twice with buffer and incubate cells in 80 μl buffers supplemented with 20 μl anti-Mouse IgG Microbeads (Miltenyi Biotec, #130-048-401, Germany) for 15 min at 4 °C. After two times of wash, cells were resuspended in 500 μl buffers and applied onto the MS column (Miltenyi, #130042-201, Germany) and collected flow-through containing unlabeled negative cells. Third, elute the magnetically labeled positive cells with 1 mL buffer and centrifugate at a speed of 300 g for 10 min at 4 °C. Cells sorted by magnetic beads were lysed immediately to obtain proteins for subsequent Western Blot. Anti-EpCAM antibodies (SANTA CRUZ, #sc-66020, USA) were used to sort epithelial cells. Anti-CD31 antibodies (SANTA CRUZ, #sc-376764, USA) were used to sort endothelial cells. Anti-CD68 antibodies (SANTA CRUZ, #sc-20068, USA) were used to sort macrophages.

Three tubes of negative cells sorted by magnetic beads in the previous step were mixed into one tube and then neutrophils were separated by centrifugation using Neutrophil Isolation Solution Kit for Various Animal Organ Tissues (Solarbio, #P2250, China) according to the density difference. According to the instructions, reagent A, reagent C and cell suspension were carefully superimposed into the centrifuge tube in turn, so that the two liquid surface interfaces between the three components were clear, and then centrifuged horizontally at 1000 g at room temperature for 30min. After centrifugation, two circular milky white cell layers would appear in the centrifuge tube, among which the lower cells were the neutrophil layer. Neutrophils were carefully absorbed through a pipette and then lysed immediately to obtain proteins for subsequent Western Blot.

6.Cell culture and hypoxia-reoxygenation (HR) model

Human primary pulmonary microvascular endothelial cells (HPMVECs) were purchased from the ScienCell Research Laboratories (#3200, USA) and cultured in Endothelial Cell Medium (ScienCell, #1001, USA). All cells were cultured in a humidified incubator at 37 °C with 5 % CO2.

To simulate clinical CPB-induced ARDS timelines, cells were subjected to hypoxia (1 % O_2_, 4 h) in Glucose-Free Endothelial Cell Medium (ScienCell, #1002, USA), followed by reoxygenation (12 h) in normal medium. After the model was established, the culture medium was collected and the cells were scraped. The culture medium was centrifuged at 1500 g at 4 °C for 15 min to obtain the supernatant, which was immediately tested for substances or frozen at −80 °C. The scraped cells were resuspended in PBS and centrifuged at 1000 rpm for 5 min at room temperature to wash thoroughly. Subsequent experiments were performed, such as substance detection, protein extraction, and nuclear and cytoplasmic separation.

7.Gene manipulation and transfection

MOTS-c overexpression plasmids, MYH9 siRNA, and scrambled controls (General Biol, China) were transfected into HPMVECs (50 % confluence) using Lipofectamine™ 3000 (Invitrogen, #L3000015, USA). Lentiviral transduction (MOI = 20) was used to express MOTS-c-GFP fusion proteins (Genechem, China). siRNA sequences are provided in Table S1.

8.Subcellular localization analysis

Cells stably expressing MOTS-c-GFP fusion protein were cultivated on glass slides and treated as required for the experiment, stained with DAPI and sealed. MOTS-c-GFP localization was visualized via fluorescence microscopy (Leica Stellaris STED DMi8). The whole cell and nuclear regions were circused in the acquired images and the fluorescence intensity was quantified using the microscope software LAS X. Six fields were selected for each slide, and the proportion of MOTS-c-GFP in the nucleus was quantified in all cells and averaged as the data of the slide sample.

Nuclear and cytoplasmic fractions were separated using a Nuclear and Cytoplasmic Protein Extraction Kit (Beyotime, #P0028, China). Briefly, the cells were sufficiently swollen and then the membrane was disrupted to release cytosolic proteins under low osmolarity conditions, followed by nuclear precipitates obtained by centrifugation. Finally, nuclear protein was extracted by high-salt nuclear protein extraction reagent. The isolated protein samples were subsequently subjected to GFP-specific Western Blot.

9.Pharmacological modulation

In cell experiments, Actinonin (150 μM) was added to the complete medium 48 h before cell modeling to deplete mitochondrial DNA and remove MOTS-c in cells, and the medium was replaced 48 h later to terminate the drug effect and the subsequent modeling was performed. *Tert*-butyl hydroperoxide (tBHP, 100 μM) was added to complete medium for continuous incubation for 16 h under normoxia to mimic ROS accumulation during HR. N-acetylcysteine (NAC, 10 mM) was added to the complete medium 2 h before modeling, and then replaced with glucose-free serum-free medium supplemented with the same concentration of NAC to establish HR model, in order to ensure that ROS could be continuously and completely scavenged at the beginning of HR and throughout the subsequent process. CK2A and PKCα inhibitors (CX4945, 10 μM; GO6983, 100 nM) were administered 2 h prior to HR modeling, and then replaced with glucose-free serum-free medium supplemented with the same concentration of inhibitors to establish HR model, in order to ensure that CK2A and PKCα could be continuously and completely inhibited at the beginning of HR and throughout the subsequent process.

In animal experiments, the synthesized MOTS-c peptide powder was dissolved in PBS and then intraperitoneally injected at 20 mg/kg/day for 5 consecutive days before the rat LIRI model was established. CX4945 (75 mg/kg) was administered by gavage and NAC (75 mg/kg) was administered by intraperitoneal injection 24 h before the rat LIRI model was established. All drugs were fully effective before modeling and no additional drugs were administered during the process of the model. Reagents were sourced from MCE and Sigma-Aldrich.

10.Endothelial barrier function assays

VE-Cadherin immunofluorescence staining was performed to visualize intercellular junctions to assess barrier morphology. Immunofluorescence staining was performed as follows. HPMVECs cultivated on glass cover slips were washed with PBS, fixed in 4 % paraformaldehyde for 15 min, and permeabilized with 0.3 % Triton X-100 for 10 min. After blocked with 10 % goat serum for 2 h at room temperature, cells were incubated with VE-Cadherin antibody (Cell Signaling Technology, #2500S, USA) overnight at 4 °C. The next day, cells were washed and incubated with Alexa Fluor® 488 goat anti-rabbit IgG (Invitrogen, #A-11008, 1:200, USA) for 2 h at room temperature, and the nuclei were stained with DAPI. Cell images were obtained using a Leica Stellaris STED DMi8 confocal laser scanning microscope.

F-actin phalloidin staining were performed to visualize cytoskeleton to assess barrier morphology. Rhodamine-labeled phalloidin dye (Solarbio, #CA1610) was incubated at room temperature for 30 min after closure and permeabilization, and the nuclei were stained with DAPI. Cell images were obtained using a Leica Stellaris STED DMi8 confocal laser scanning microscope.

*Trans*-endothelial electric resistance (TEER) is a direct indicator of the monolayer's resistance to the passage of electrical currents and provide a real-time assessment of cellular barrier function during HR. HPMVECs were cultivated in transwell chambers and grown to nearly 100 % confluence. The resistance between the upper and lower layers of the chamber was measured by Millipore Millicell ERS-2 cell resistance meter, and the normalized TEER was calculated by subtracting the resistance measured in the chamber containing only medium without seeded cells. The test was repeated 2 h after the first measurement, and only when the results were not statistically different from the previous results, the subsequent modeling experiment could be carried out to ensure the stability of the current barrier function. TEER was not detected during 4 h of hypoxia to avoid interfering with the hypoxic process. The medium was changed immediately after the end of hypoxia, and TEER was measured once. During the subsequent 12 h of reoxygenation, measurements were made every 2 h for a total of six times. Six replicates were set for each differently treated cell group. Each time point was measured three times and averaged. Line graphs were drawn using the data of nine time points, and the normalized TEER at the last time point (16 h of reoxygenation) was used for statistical analysis.

The dextran leakage assay is a classical method to assess the void in monolayer cells. Cells were cultivated on glass slides to obtain representative fluorescence images or cultivated in chambers to quantify the amount of fluorescent dye leakage. When confluence was close to 100 %, pharmacological and modeling treatments were performed according to experimental requirements. Then cells were incubated with FITC-labeled dextran (1 mg/mL) for 30 min under normal culture conditions, during which the fluorescent dye crossed the intercellular space into the bottom surface of the cells or the lower layer of the chambers. The glass slides were gently cleaned and sealed, and cell images were obtained using Leica THUNDER Imager microscope. The fluorescent solution from the lower layer of the chambers was collected and the fluorescence intensity at 488 nm was measured using a Biotech Synergy H1 Microplate Reader.

11.Oxidative stress evaluation

Intracellular reactive oxygen species (ROS) accumulation, as a direct indicator of oxidative stress, were quantified using DCFH-DA fluorescence (Beyotime, #S0033S, China) via microscopy, microplate reader or flow cytometry.

Lipid peroxidation and glutathione ratios were measured using Lipid Peroxidation MDA Assay Kit (Beyotime, #S0131S, China) and GSH and GSSG Assay Kit (Beyotime, #S0053, China) according to the manufacturer's instructions. Samples of various tissue sources were processed for each assay as follows. The lung tissues were homogenized and dissolved in extraction buffer. Rat plasma and human serum were immediately centrifuged at 1500 g 4 °C after collection, and supernatants were separated and frozen at −80 °C until measured.

12.Western Blot

Lung tissue samples or cells were lysed in RIPA buffer (Beyotime, #P0013B, China) with PMSF (Beyotime, #ST505, China) for 20 min. The protein concentrations were measured using a BCA protein assay kit (Beyotime, #P0010, China), and 50 μg of proteins were transferred onto a PVDF membrane following separation on a 10 % SDS-polyacrylamide gel. The membrane was blocked with 5 % (w/v) nonfat dry milk for 2 h, followed by an overnight incubation at 4 °C with corresponding primary antibody. Subsequently, the membrane was incubated for an additional 1 h with HRP-conjugated secondary antibody (1:10000 dilution) at room temperature after thoroughly washing three times with TBST. Bands were detected by ECL (Beyotime, #P0018AM, China) and band intensities were quantified using Image J gel analysis software.

Antibodies to MOTS-c (#MOTSC-101AP) were provided by Thermo Fisher Fabgennix (USA). Antibodies to Phospho-Myosin IIa (Ser1943) (#14611S) were provided by Cell Signaling Technology (USA). Antibodies to HIF1A (# 20960-1-AP), GFP (#66002-1-Ig), MYH9 (#11128-1-AP), HMOX1 (#10701-1-AP), NQO1 (#11451-1-AP), PRDX6 (13585-1-AP), Lamin B1 (#12987-1-AP) and β-Actin (66009-1-Ig) were provided by Proteintech (China). GPX2 (#ab137431), γ-Actin (ab123034) were provided by Abcam (UK). antibodies to COX1 (#sc-19998) were kindly provided by SANTA CRUZ (USA).

13.Enzyme linked immunosorbent assay (ELISA)

MOTS-c and IL-6 concentrations in human serum, rat plasma, and cell culture medium were measured using EIAab (#E15563h), CLOUD CLONE (#CEX132Ra), and MULTI SCIENCES (#EK106HS, #EK306HS) kits, respectively. Absorbance was read at 450 nm using Multiskan SkyHigh Microplate Reader (Thermo Fisher, USA). All assays were performed in accordance with manufacturer's protocols.

14.Quantitative Reverse Transcription Polymerase Chain Reaction

Analysis of MTRNR1, COX1, MYH9, HIF1A, HMOX1, NQO1, GPX2, PRDX6, and IL6 mRNA expressions of cells or rat lung tissues were performed by quantitative real-time reverse transcription polymerase chain reaction (RT-qPCR). Total RNA was extracted using the RNAeasy™ Animal RNA Isolation Kit with Spin Column (Beyotime, #R0027, China), and reverse-transcribed into cDNA using SweScript RT II First Strand cDNA Synthesis Kit (Servicebio, #G3333-50, China). RT-qPCR was performed using ChamQ Universal SYBR qPCR Master Mix (Vazyme, #Q711-02, China) using gene-specific primers (Table S2). The amplification cycling reactions (40 cycles) were as follows: 10s at 95 °C and 30s at 60 °C. Relative mRNA expression was calculated using the 2^−ΔΔCT^ method. The primer sequences are provided in Table S2.

15.Immunoprecipitation assay (IP)

Protein interactions were validated via immunoprecipitation using Pierce MS-Compatible Magnetic IP Kit (Protein A/G) (Thermo Scientific, #90409, USA). All assays were performed in accordance with manufacturer's protocols. In brief, cells were lysed with ice-cold IP-MS Cell Lysis Buffer. The lysates were incubated with the corresponding primary antibody or control IgG overnight on a rotating shaker at 4 °C, followed by the addition of Protein A/G Magnetic Beads for another 2 h. Proteins were eluted by IP-MS Elution Buffer, followed by mass spectrometry or Western Blot as described above.

16.Chromatin immunoprecipitation (ChIP)

Direct interaction between MOTS-c and chromatin was explored via chromatin immunoprecipitation Pierce Magnetic ChIP Kit (Thermo Scientific, #26157, USA). Briefly, the cells were fixed with 1 % formaldehyde at room temperature for 10 min. Cross-linking reactions were quenched by the addition of glycine. The cells were then washed with ice-cold 1 × PBS, and after collection, they were treated with Membrane Extraction Buffer and MNase Digestion Buffer and then sonicated to obtain the 200–1000 bp DNA fragments. The sheared chromatin was immunoprecipitated with the GFP tag antibody, Anti-RNA Polymerase II Antibody or Normal Rabbit IgG at 4 °C overnight, followed by the addition of ChIP Grade Protein A/G Magnetic Beads for another 2 h. The beads were resuspended after several washes and heated at 65 °C for 30 min with vigorous shaking. NaCl and Proteinase K were added after the tubes had been cooled on ice. The tubes were then incubated in a 65 °C heat block for 1.5 h. After DNA recovery, the finally eluted solution containing purified DNA was collected to perform sequencing or RT-qPCR analysis, and the fold enrichment relative to the IgG control antibody was calculated.

17.Luciferase reporter assays

HPMVEC cells were transfected with pIL-6-promoter-luc (Beyotime, #D2386, China) or Cignal Antioxidant Luciferase Reporter Kit (Qiagen, #CCS-5020L, USA) and Lipofectamine™ 3000 Reagent (Invitrogen, #L3000015, USA). Luciferase activity was measured using Firefly Luciferase Reporter Gene Assay Kit (Beyotime, #RG005, China).

18.Molecular docking

Use the GRAMM (http://gramm.compbio.ku.edu/) complete MOTS-c with MYH9-γ-Actin molecular docking. The crystal structures of human MOTS-c, MYH9, and γ-Actin, were predicted by AlphaFold. Firstly, rigid protein docking was performed between MYH9 protein and γ-Actin protein to construct a protein dimer (MYH9-γ-Actin). Then, protein-polypeptide docking analysis was performed between MYH9-γ-Actin and MOTS-c using GRAMM to study the interaction between them. Finally, Pymol v2.6 software was used to draw 3D images.

19.Synthesis of MOTS-c and in vivo administration

MOTS-c peptide (MRWQEMGYIFYPRKLR) was synthesized by QYAOBIO (China) (purity >97 %). The synthesized MOTS-c peptide powder was dissolved in PBS and then intraperitoneally injected at 20 mg/kg/day for 5 consecutive days, and then processed or modeled according to experimental requirements.

20.Statistical analysis

All statistical analyses were conducted using GraphPad Prism (version 10.0) and R software (version 4.2.1). Continuous variables were expressed as median with interquartile range (IQR) or mean ± standard deviation (SD), depending on data distribution assessed by Shapiro-Wilk test. Between-group comparisons for two independent samples were performed using unpaired two-tailed Student's t-test for normally distributed data or Mann-Whitney *U* test for non-parametric data. For comparisons involving three or more groups, one-way ANOVA with Tukey's post hoc test or Kruskal-Wallis test with Dunn's correction was applied as appropriate. To evaluate the predictive capacity of MOTS-c dynamics for ARDS development, receiver operating characteristic (ROC) curves were generated, and area under the curve (AUC) values with 95 % confidence intervals (CI) were calculated. Model performance comparisons between classical and modified predictive models (incorporating ΔMOTS-c) were assessed using the Delong test. Integrated discrimination improvement (IDI) and category-free net reclassification improvement (cfNRI) were computed to quantify classification accuracy enhancements. Multivariable logistic regression models adjusted for perioperative characteristics were constructed to identify independent predictors of ARDS, with results reported as odds ratios (OR) and 95 % CI. Calibration of predictive models was validated via the Hosmer-Lemeshow goodness-of-fit test. Survival analysis in animal studies utilized Kaplan-Meier curves, and differences between groups were evaluated using the log-rank test.

Experimental data for each quantitative analysis were replicated at least three times. Statistical significance was defined as ∗P < 0.05, ∗∗P < 0.01, ∗∗∗P < 0.001, ∗∗∗∗P < 0.0001.

## CRediT authorship contribution statement

**Xiangyu Li:** Writing – original draft, Investigation, Conceptualization. **Faliang Zhan:** Writing – review & editing, Conceptualization. **Guangfeng Qiu:** Writing – review & editing, Conceptualization. **Peng Lu:** Investigation. **Zihao Shen:** Investigation. **Yuanpu Qi:** Investigation. **Minchao Wu:** Investigation. **Mingyu Chu:** Investigation. **Jia Feng:** Investigation. **Ziang Wen:** Methodology. **Xin Yao:** Methodology. **Ao Wang:** Methodology. **Wanjun Jin:** Writing – original draft. **Xiao Zhang:** Writing – original draft. **Junjie Liao:** Writing – original draft. **Jialin Zhang:** Writing – original draft. **Meijuan Song:** Writing – review & editing, Funding acquisition, Conceptualization. **Wei Wang:** Writing – review & editing, Conceptualization. **Xiaowei Wang:** Writing – review & editing, Funding acquisition, Conceptualization.

## Informed consent statement

Patient consent was waived due to the retrospective nature of the study.

## Clinical trial number

Not applicable.

## Institutional review board statement

The study was conducted in accordance with the Declaration of Helsinki and approved by the Ethics Committee of The First Affiliated Hospital with Nanjing Medical University (protocol code 2023-SR-075).

## 6 Funding

This research was supported by the 10.13039/501100001809National Natural Science Foundation of China (NO. 82470419), the Key Project of Taizhou School of Clinical Medicine (NO. TZKY20220304), the Yili & Jiangsu Joint Institute of Health (NO. YL2022MS04), the Science Foundation Project of Yili & Jiangsu Joint Institute of Health (NO. ly2023zd01), and the 2023rd First Affiliated Hospital with Nanjing Medical University's National Natural Science Foundation of China Youth Fund Cultivation Program (NO. PY2023022).

## Declaration of competing interest

The authors declare no conflicts of interest.

## Figures and Tables

**Supplementary Fig. 4:**
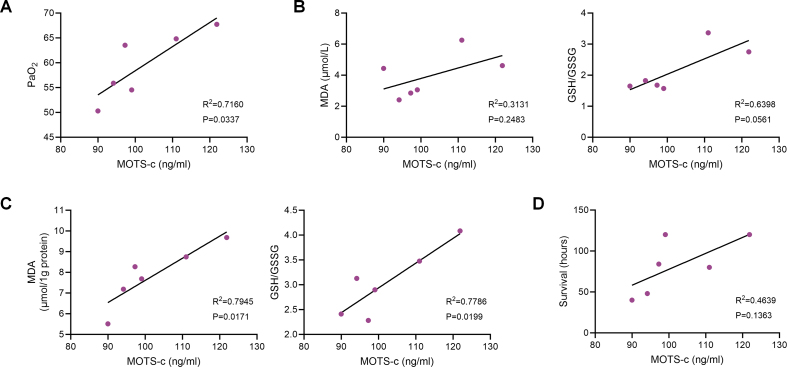
Correlation analysis of plasma MOTS-c levels in the rat LIRI model **(A)** Scatter plot and linear regression analysis of plasma MOTS-c concentration versus PaO_2_ at the endpoint of LIRI modeling (n = 6, simple linear regression). **(B)** Scatter plot and linear regression analysis of plasma MOTS-c levels correlated with plasma malondialdehyde (MDA) and GSH/GSSG ratios (n = 6, simple linear regression). **(C)** Scatter plot and linear regression analysis of plasma MOTS-c levels correlated with MDA and GSH/GSSG ratios in whole-lung tissue lysates (n = 6, simple linear regression). **(D)** Scatter plot and linear regression analysis of plasma MOTS-c concentration versus 5-day survival duration in LIRI rats (n = 6, simple linear regression). Abbreviations: PaO_2_, arterial oxygen tension; MDA, malondialdehyde; GSH, reduced glutathione; GSSG, oxidized glutathione.

## Data Availability

Data will be made available on request.

## References

[1] Bartoszko J., Karkouti K. (2021). Managing the coagulopathy associated with cardiopulmonary bypass. J. Thromb. Haemostasis.

[2] Kamenshchikov N.O., Anfinogenova Y.J., Kozlov B.N. (2022). Nitric oxide delivery during cardiopulmonary bypass reduces acute kidney injury: a randomized trial. J. Thorac. Cardiovasc. Surg..

[3] Zhou L., Liu X., Yan M. (2021). Postoperative Nadir hemoglobin and adverse outcomes in patients undergoing on-pump cardiac operation. Ann. Thorac. Surg..

[4] Wang Y., Chen L., Yao C. (2023). Early plasma proteomic biomarkers and prediction model of acute respiratory distress syndrome after cardiopulmonary bypass: a prospective nested cohort study. Int. J. Surg..

[5] Rong L.Q., Di Franco A., Gaudino M. (2016). Acute respiratory distress syndrome after cardiac surgery. J. Thorac. Dis..

[6] García-Delgado M., Navarrete-Sánchez I., Colmenero M. (2014). Preventing and managing perioperative pulmonary complications following cardiac surgery. Curr. Opin. Anaesthesiol..

[7] Damgaard S., Nielsen C.H., Andersen L.W. (2010). Cell saver for on-pump coronary operations reduces systemic inflammatory markers: a randomized trial. Ann. Thorac. Surg..

[8] Warren O., Alexiou C., Massey R. (2007). The effects of various leukocyte filtration strategies in cardiac surgery. Eur. J. Cardio. Thorac. Surg..

[9] Young R.W. (2014). Prevention of lung injury in cardiac surgery: a review. J. Extra Corpor. Technol..

[10] Needham D.M., Korupolu R., Zanni J.M. (2010). Early physical medicine and rehabilitation for patients with acute respiratory failure: a quality improvement project. Arch. Phys. Med. Rehabil..

[11] Pasquina P., Tramèr M.R., Walder B. (2003). Prophylactic respiratory physiotherapy after cardiac surgery: systematic review. BMJ.

[12] Al-Sarraf N., Thalib L., Hughes A. (2008). Effect of smoking on short-term outcome of patients undergoing coronary artery bypass surgery. Ann. Thorac. Surg..

[13] Myers K., Hajek P., Hinds C. (2011). Stopping smoking shortly before surgery and postoperative complications: a systematic review and meta-analysis. Arch. Intern. Med..

[14] Mehta C., Mehta Y. (2016). Management of refractory hypoxemia. Ann. Card Anaesth..

[15] Peek G.J., Mugford M., Tiruvoipati R. (2009). Efficacy and economic assessment of conventional ventilatory support versus extracorporeal membrane oxygenation for severe adult respiratory failure (CESAR): a multicentre randomised controlled trial. Lancet.

[16] Li Y., Li Z., Ren Y. (2024). Mitochondrial-derived peptides in cardiovascular disease: novel insights and therapeutic opportunities. J. Adv. Res..

[17] Lee C., Zeng J., Drew B.G. (2015). The mitochondrial-derived peptide MOTS-c promotes metabolic homeostasis and reduces obesity and insulin resistance. Cell Metab..

[18] Zhang Y., Huang J., Li S. (2024). Pyrroloquinoline quinone alleviates mitochondria damage in radiation-induced lung injury in a MOTS-c-dependent manner. J. Agric. Food Chem..

[19] Lu H., Fan L., Zhang W. (2024). The mitochondrial genome-encoded peptide MOTS-c interacts with Bcl-2 to alleviate nonalcoholic steatohepatitis progression. Cell Rep..

[20] Lin C., Luo L., Xun Z. (2024). Novel function of MOTS-c in mitochondrial remodelling contributes to its antiviral role during HBV infection. Gut.

[21] Wang D., Xu B., Sun J. (2025). MOTS-c mimics remote ischemic preconditioning in protecting against lung ischemia-reperfusion injury by alleviating endothelial barrier dysfunction. Free Radic. Biol. Med..

[22] Yaşar E., Çakmak T., Bayramoğlu A. (2022). MOTS-c as a predictor of coronary lesions and complexity in patients with stable coronary artery disease. Eur. Rev. Med. Pharmaco..

[23] Goetzl E.J., Wolkowitz O.M., Srihari V.H. (2021). Abnormal levels of mitochondrial proteins in plasma neuronal extracellular vesicles in major depressive disorder. Mol. Psychiatr..

[24] Domin R., Pytka M., Żołyński M. (2023). MOTS-C serum concentration positively correlates with lower-body muscle strength and is not related to maximal oxygen uptake-A preliminary study. Int. J. Mol. Sci..

[25] Kim K.H., Son J.M., Benayoun B.A. (2018). The mitochondrial-encoded peptide MOTS-c translocates to the nucleus to regulate nuclear gene expression in response to metabolic stress. Cell Metab..

[26] Vicente-Manzanares M., Ma X., Adelstein R.S. (2009). Non-muscle myosin II takes centre stage in cell adhesion and migration. Nat. Rev. Mol. Cell Biol..

[27] Moussavi R.S., Kelley C.A., Adelstein R.S. (1993). Phosphorylation of vertebrate nonmuscle and smooth muscle myosin heavy chains and light chains. Mol. Cell. Biochem..

[28] Dulyaninova N.G., Bresnick A.R. (2013). The heavy chain has its day: regulation of myosin-II assembly. BioArchitecture.

[29] Sanborn K.B., Mace E.M., Rak G.D. (2011). Phosphorylation of the myosin IIA tailpiece regulates single myosin IIA molecule association with lytic granules to promote NK-cell cytotoxicity. Blood.

[30] Ludowyke R.I., Elgundi Z., Kranenburg T. (2006). Phosphorylation of nonmuscle myosin heavy chain IIA on Ser1917 is mediated by protein kinase CβII and coincides with the onset of stimulated degranulation of RBL-2H3 mast cells. J. Immunol..

[31] Conti M.A., Sellers J.R., Adelstein R.S. (1991). Identification of the serine residue phosphorylated by protein kinase C in vertebrate nonmuscle myosin heavy chains. Biochemistry.

[32] D W., Dj J. (2009). Protein kinase CK2 regulates cytoskeletal reorganization during ionizing radiation-induced senescence of human mesenchymal stem cells. Cancer Res..

[33] K K., R K., R B. (2006). Signaling via the angiotensin-converting enzyme results in the phosphorylation of the nonmuscle myosin heavy chain IIA. Mol. Pharmacol..

[34] Waters C.M., Bassler B.L. (2005). Quorum sensing: cell-to-cell communication in bacteria. Annu. Rev. Cell Dev. Biol..

[35] Jovaisaite V., Auwerx J. (2015). The mitochondrial unfolded protein response—synchronizing genomes. Curr. Opin. Cell Biol..

[36] Galluzzi L., Kepp O., Kroemer G. (2012). Mitochondria: master regulators of danger signalling. Nat. Rev. Mol. Cell Biol..

[37] Mercer T.R., Neph S., Dinger M.E. (2011). The human mitochondrial transcriptome. Cell.

[38] Niu F., Li L., Wang L. (2024). Autoinhibition and activation of myosin VI revealed by its cryo-EM structure. Nat. Commun..

[39] Jans D.A., Hübner S. (1996). Regulation of protein transport to the nucleus: central role of phosphorylation. Physiol. Rev..

[40] Sun Z., Ning Y., Wu H. (2023). 14-3-3ζ targets β-catenin nuclear translocation to maintain mitochondrial homeostasis and promote the balance between proliferation and apoptosis in cisplatin-induced acute kidney injury. Cell. Signal..

[41] Yang L., Xiao J.-J., Zhang L. (2025). Methionine sulfoxide reductase A deficiency aggravated ferroptosis in LPS-induced acute kidney injury by inhibiting the AMPK/NRF2 axis and activating the CaMKII/HIF-1α pathway. Free Radic. Biol. Med..

[42] Kalukula Y., Stephens A.D., Lammerding J. (2022). Mechanics and functional consequences of nuclear deformations. Nat. Rev. Mol. Cell Biol..

[43] Dieleman J.M., Nierich A.P., Rosseel P.M. (2012). Intraoperative high-dose dexamethasone for cardiac surgery: a randomized controlled trial. JAMA.

[44] Eren N., Cakir O., Oruc A. (2003). Effects of N-acetylcysteine on pulmonary function in patients undergoing coronary artery bypass surgery with cardiopulmonary bypass. Perfusion.

