# Supplementary material for: MOTS-c attenuates lung ischemia-reperfusion injury via MYH9-Dependent nuclear translocation and transcriptional activation of antioxidant genes
Source: Redox Biol. 2025 May 15;84:103681. doi: 10.1016/j.redox.2025.103681 (PMC12150175; doi:10.1016/j.redox.2025.103681)
Supplement: Multimedia component 1 [file mmc1.docx]

**Table S1. The sequences of the siRNA oligos used for MYH9 knockdown**

| MYH9-1 | 5'-GCAAGCUGCCGAUAAGUAUTT-3' |
| --- | --- |
| MYH9-2 | 5'-CCAGAAGGCGCAGACUAAATT-3' |
| MYH9-3 | 5'-CCAGUCCUCUGACAAGUUUTT-3' |
