# Supplementary material for: MOTS-c attenuates lung ischemia-reperfusion injury via MYH9-Dependent nuclear translocation and transcriptional activation of antioxidant genes
Source: Redox Biol. 2025 May 15;84:103681. doi: 10.1016/j.redox.2025.103681 (PMC12150175; doi:10.1016/j.redox.2025.103681)
Supplement: Multimedia component 2 [file mmc2.docx]

**Table S2. The sequences of the primer used for RT-qPCR**

| human MTRNR1 | F：5'-ATACCGCCATCTTCA-3'  R：5'-ATCCTCCTTAGTCCTTT-3' |
| --- | --- |
| human COX1 | F：5'-CTTTTCACCGTAGGTGGCCT-3'  R：5'-AGTGGAAGTGGGCTACAACG-3' |
| human MYH9 | F：5'-CCTCAAGGAGCGTTACTACTCA-3'  R：5'-CTGTAGGCGGTGTCTGTGAT-3' |
| human HIF1A | F：5'-GAACGTCGAAAAGAAAAGTCTCG-3'  R：5'-CCTTATCAAGATGCGAACTCACA-3' |
| human HMOX1 | F：5'-CTCAAACCTCCAAAAGCC-3'  R：5'-TCAAAAACCACCCCAACCC-3' |
| human NQO1 | F：5'-TGCAGCGGCTTTGAAGAAGAAAGG-3'  R：5'-TCGGCAGGATACTGAAAGTTCGCA-3' |
| human GPX2 | F：5'-GGTAGATTTCAATACGTTCCGGG-3'  R：5'-TGACAGTTCTCCTGATGTCCAAA-3' |
| human PRDX6 | F：5'-CAGTGTGCACCACAGAGCTT-3'  R：5'-CAAGCTCCCGATTCCTATCA-3' |
| human IL6 | F：5'-GTAGCCGCCCCACAGA-3'  R：5'-CATGTCTCCTTTCTCAGGGCTG-3' |
| human RN18S | F：5'-TTAATTCCGATAACGAACGAGA-3'  R：5'-CGCTGAGCCAGTCAGTGTAG-3' |
| rat HIF1A | F：5'-GCGGCGAGAACGAGAAGAAA-3'  R：5'-AGATGGGAGCTCACGTTGTG-3' |
| rat HMOX1 | F：5'-CAGTCTATGCCCCGCTCTAC-3'  R：5'-ACCAGCAGCTCAGGATGAGT-3' |
| rat NQO1 | F：5'-GAGCCCGGATATTGTAGCTG-3'  R：5'-CTTCTTATTCTGGAAAGGACCC-3' |
| rat GPX2 | F：5'-GACACGAGGAAACCGAAGCA-3'  R：5'-GGCCCTTCACAACGTCT-3' |
| rat PRDX6 | F：5'-ACAGCCCGTGTGGTATTCAT-3'  R：5'-CTCTCTCCCTTCTTCCAGTCAA-3' |
| rat RN18S | F：5'-GCTGAGAAGACGGTCGAACT-3'  R：5'-TTAATGATCCTTCCGCAGGT-3' |
